# Cryptic Chemical Variation in a Marine Red Alga as Revealed by Nontargeted Metabolomics

**DOI:** 10.1021/acsomega.3c00301

**Published:** 2023-04-06

**Authors:** Bhuwan Khatri Chhetri, Nazia Mojib, Samuel G. Moore, David A. Delgadillo, Jessica E. Burch, Nolan H. Barrett, David A. Gaul, Lewis Marquez, Katy Soapi, Hosea M. Nelson, Cassandra L. Quave, Julia Kubanek

**Affiliations:** † School of Chemistry and Biochemistry, Georgia Institute of Technology, Atlanta, Georgia 30332, United States; ‡ Center for Microbial Dynamics and Infection, Georgia Institute of Technology, Atlanta, Georgia 30332, United States; § School of Biological Sciences, Georgia Institute of Technology, Atlanta, Georgia 30332, United States; @ Department of Biology, Spelman College, Atlanta, Georgia 30314, United States; ∇ Department of Dermatology, Center for the Study of Human Health, and Antibiotic Resistance Center, Emory University, Atlanta, Georgia 30322, United States; ξ Institute of Applied Sciences, University of South Pacific, Suva, Fiji; Θ Parker H. Petit Institute for Bioengineering and Bioscience, Georgia Institute of Technology, Atlanta, Georgia 30332, United States; δ School of Earth and Atmospheric Sciences, Atlanta, Georgia 30332, United States; # Division of Chemistry and Chemical Engineering, California Institute of Technology, Pasadena, California 91125, United States

## Abstract

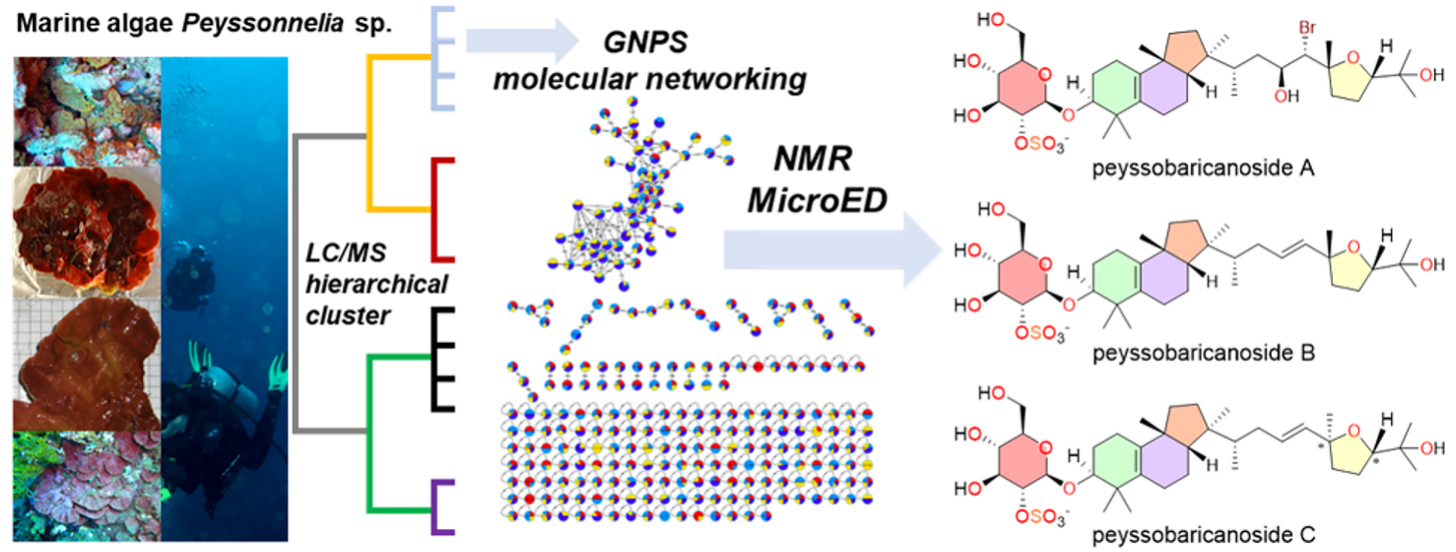

Many marine algae occupy habitats that are dark, deep, or encrusted on other organisms and hence are frequently overlooked by natural product chemists. However, exploration of less-studied organisms can lead to new opportunities for drug discovery. Genetic variation at the individual, species, genus, and population levels as well as environmental influences on gene expression enable expansion of the chemical repertoire associated with a taxonomic group, enabling natural product exploration using innovative analytical methods. A nontargeted LC-MS and ^1^H NMR spectroscopy-based metabolomic study of 32 collections of representatives of the calcareous red algal genus *Peyssonnelia* from coral reef habitats in Fiji and the Solomon Islands revealed significant correlations between natural products’ chemistry, phylogeny, and biomedically relevant biological activity. Hierarchical cluster analysis (HCA) of LC-MS data in conjunction with NMR profiling and MS/MS-based molecular networking revealed the presence of at least four distinct algal chemotypes within the genus *Peyssonnelia*. Two Fijian collections were prioritized for further analysis, leading to the isolation of three novel sulfated triterpene glycosides with a rearranged isomalabaricane carbon skeleton, guided by the metabolomic data. The discovery of peyssobaricanosides A–C (**15**–**17**) from two Fijian *Peyssonnelia* collections, but not from closely related specimens collected in the Solomon Islands that were otherwise chemically and phylogenetically very similar, alludes to population-level variation in secondary metabolite production. Our study reinforces the significance of exploring unusual ecological niches and showcases marine red algae as a chemically rich treasure trove.

## Introduction

While many have shown that genes predict phenotype, it is also clear that the environment interacts unexpectedly with genetics to drive gene expression, protein translation, and finally biosynthesis of metabolites which are the ultimate products of living systems. Metabolomics has the potential to define the vast universe of chemical diversity arising from metabolic activity among millions of unique organisms. Metabolic responses of living cells to disease, to other cells, and to their environment act as multipliers of existing genetic diversity, further expanding the chemical diversity of Earth’s global metabolome. Comprehensive access to this chemical diversity can serve as inspiration for cures for diseases, promote solutions to environmental threats, and lead to a predictive understanding of the rules that govern biology at the chemical level. In effect, metabolites are the words to a complex—if so far only partly deciphered—language of life on Earth.

The marine red algal genus *Peyssonnelia* is globally distributed in tropical to warm temperate habitats, forming an integral part of many marine reef ecosystems.^1,2^*Peyssonnelia* is the most species-rich genus in the family Peyssonneliaceae with 89 currently recognized species.^3^ Along with other calcareous algae, they deposit calcium carbonate, bind adjacent substrata, and act as a barrier against erosion, hence contributing to the overall construction and maintenance of the reef framework. The calcareous surface of *Peyssonnelia* has been found to facilitate the settlement and metamorphosis of coral larvae, which in turn aids the buildup of coral reefs.^4^

In tropical coral reef environments, micro- and macroorganisms including *Peyssonnelia* spp. face strong pressure for survival and reproduction. As reef ecosystems are space-limited yet species-diverse, *Peyssonnelia* spp. are often found growing in proximity with other encrusting algae and invertebrates, resulting in competition for light, substrate, and nutrients. In addition, benthic organisms are frequently confronted by voracious marine grazers such as herbivorous fishes and sea urchins, as well as by disease-causing microorganisms. Notably, a microbial infection called *Peyssonnelia* Yellow Band Disease (PYBS) has detrimental effects on *Peyssonnelia* spp.^5^ Hence, members of the *Peyssonnelia* genus, like other benthic species in coral reef settings, face intense pressure to evolve resistance to various threats from disease-causing microorganisms, grazers, and competitors.

Production of defensive secondary metabolites is an important mechanism employed by many sessile marine organisms as deterrence against enemies such as predators and pathogens.^6^ Additionally, as *Peyssonnelia* spp. thrive in challenging underwater terrains composed of caves and crevices with poor light penetration and thus limited photosynthesis, the replacement of damaged algal tissue may in fact be especially metabolically expensive, increasing the value of chemical defenses that protect tissue from attack. The secondary metabolism of *Peyssonnelia* spp. is not well studied, with reports of only 14 natural products representing five structural classes to date.^7−11^ This lack of attention by natural product chemists can be partly attributed to the encrusting nature of *Peyssonnelia* spp. along with the difficult habitats that these species inhabit, making specimens challenging to collect in bulk. Finally, intraspecific phenotypic variation and overlapping morphological traits among the many *Peyssonnelia* species make field-based identification perplexing even for scientists with training in algal taxonomy, lending to this genus a sense of crypsis, whereby morphology does not appear to predict chemistry. Nevertheless, the previously described molecules from *Peyssonnelia* belong to diverse biosynthetic classes, represent unusual carbon skeletons, and show promising biological activities relevant to human disease.^7,9−11^ Taken together, we hypothesize that less-studied algae like those represented by the genus *Peyssonnelia*, with rich genetic and morphological diversity, are strong candidates for exploration of secondary metabolism which may be further diversified by the unusual ecological niches where they thrive.

As a tool for uncovering cryptic variation in secondary metabolism, nontargeted metabolomics has been successfully applied to study natural product diversity and to prioritize for more detailed study specimens that are unique in their secondary metabolite profiles.^12−14^ Nontargeted metabolomics may be especially useful for exploration of less-studied organisms such as *Peyssonnelia* spp., enabling classification of specimens according to similar chemical profiles which can be exploited to uncover rare genetic variants of specimens that are phenotypically indistinguishable. Prioritization of specimens using metabolomics is gradually becoming established as a critical step in drug discovery,^12^ whereby conventional bioassay-guided fractionation often leads to rediscovery of known compounds. Finally, effects of habitat and ecological interactions on gene expression of secondary metabolites can also be captured by metabolomic analyses of specimens occupying variable ecological niches. Herein, we apply nontargeted metabolomics analyses combining MS and NMR spectroscopy to reveal the promise of natural products from marine red algae of the genus *Peyssonnelia*.

## Results and Discussion

**Figure 1:**
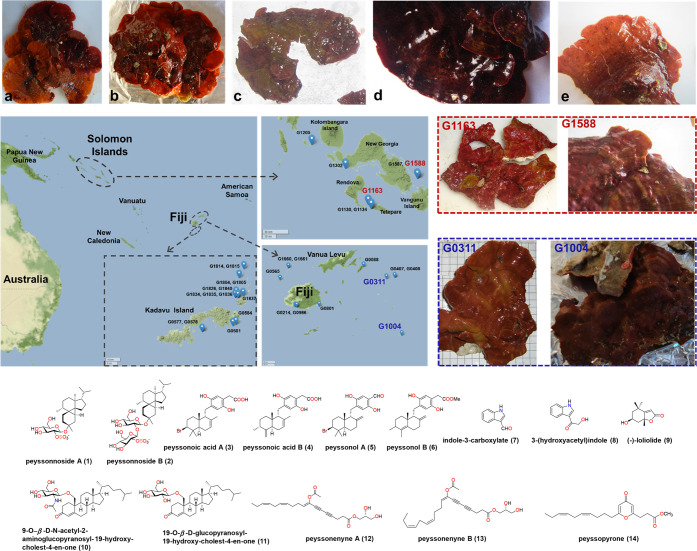
Peyssonnelia ssp. algae samples and natural products. Top: Photos from time of collection depicting the variable morphologies of various Peyssonnelia spp. from Fiji and the Solomon Islands: Collections (a) G1205, (b) G0986, (c) G0581, (d) G0584, and (e) G1587. Middle left: Collection sites for the 32 Peyssonnelia spp. samples. Middle right: Calcified morphology of prioritized Peyssonnelia spp. collections from the Solomon Islands (G01163, G1588; red outline) and Fiji (G0311, and G1004; blue outline). Bottom: Natural products previously reported from red algae of the genus Peyssonnelia.^7−11^ Photographs courtesy of M.E. Hay. Copyright 2023.

To interrogate the chemical diversity and bioactivity harbored by red algae of the genus *Peyssonnelia*, 32 collections from coral reef habitats in Fiji and the Solomon Islands were selected, representing a range of morphological traits. The most apparent feature that differentiated the specimens was their degree of calcification: some collections had a leafy, paper-like, or leathery consistency, whereas others had a crunchy, potato chip, or cement-like texture that depended on the extent of calcification (Figure 1, Figure S1). Other differences included variable color, tissue thickness, blade size, and extent to which the alga was encrusted on other organisms. Based on the substantial morphological variation observed among our 32 collections, we speculated that our sample set represented multiple *Peyssonnelia* species and populations, enabling insight into the underlying chemical diversity.

The organic extract of each *Peyssonnelia* collection was subjected to reversed phase chromatographic separation with HP20SS to obtain four fractions (A–D), in decreasing order of polarity. Bioassay and metabolomic analysis were conducted with the two midpolarity fractions (B and C) that were devoid of inorganic salts and the most highly nonpolar lipids. The most promising antimicrobial activities against methicillin-resistant *Staphylococcus aureus* (MRSA) were associated with collections G0311, G0408, G0581, G0584, G1004, G1163, and G1588 (Table 1). The midpolarity fraction C (methanol-soluble) of G1205 (>97% inhibition of parasite growth at a test concentration of 8.3 μg/mL) was one of the most active hits from our entire extract library against liver stage malaria (*Plasmodium falciparum*). Although not explored further in the present study because of limited sample availability, midpolarity fraction C of *Peyssonnelia* collection G0631 showed strong growth inhibition of amphotericin-B-resistant *Candida albicans* (ARCA, MIC_90_ 2.0 μg/mL). Hence, the promising bioactivity observed with selected *Peyssonnelia* specimens against a variety of disease targets illustrated the biomedical potential of this less-studied genus. The bioassay data presented here along with bioactivities previously reported in the literature are associated with only a small subset of the total chemical diversity harbored by members of the genus *Peyssonnelia*.

**Table 1:** Mid-polarity Fractions Obtained from 32 Peyssonnelia Collections Showing Most Promising Antimicrobial Activity against Methicillin-Resistant Staphylococcus aureus (MRSA)^a^

Sample	MIC (μg/mL)
G0584_B	3.2 ± 0.3
G0584_C	25 ± 2
G0581_B	2.7 ± 0.7
G0581_C	4 ± 2
G0408_B	7.3 ± 0.2
G0408_C	11.6 ± 0.7
G01163_B	14.2 ± 0.2
G1588_B	38 ± 4
G0311_B	59 ± 1
G0311_C	46 ± 4
G1004_C	41 ± 4

a B: eluted from HP-20SS with 80% aqueous methanol, C: eluted with 100% methanol. Fractions not shown in Table 1 (fractions B and C for the remainder of the 32 *Peyssonnelia* collections) were tested but found to be inactive at the highest test concentration of 125 μg/mL. Fractions A and D (50% aqueous methanol and 100% acetone-eluting fractions, respectively) were not subjected to bioassay as they primarily contained salts, lipids, and algal pigments.

**Figure 2:**
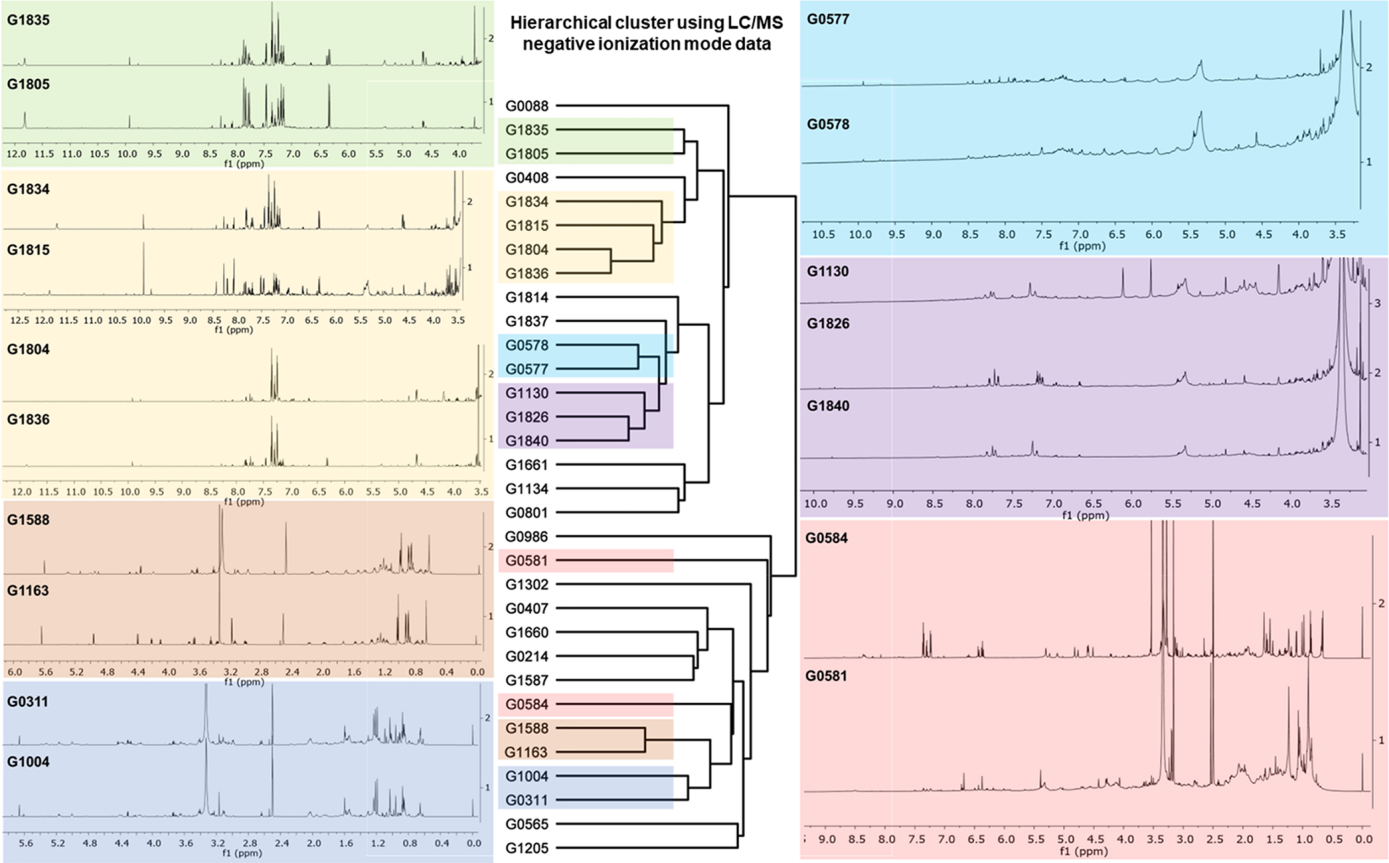
^1^H NMR spectra (left, right) and LC-MS based HCA (middle) for midpolarity HP20SS fraction B from 32 *Peyssonnelia* collections complement each other, illuminating the chemical diversity among *Peyssonnelia* spp. sampled in Fiji and the Solomon Islands.

Hierarchical cluster analysis (HCA) of the negative ionization mode LC-MS data in conjunction with ^1^H NMR spectroscopic data revealed rich chemical diversity within the 32 *Peyssonnelia* collections (Figure 2). Not surprisingly, peyssonnoside A (**1**)-containing samples, G1163 and G1588, collected from reefs within 100 km of each other in the Solomon Island shared many morphological features (Figure 1) and clustered together by LC-MS based HCA. Unfortunately, phylogenetic comparison of these two samples using 18S rRNA was not feasible as multiple efforts to extract and amplify DNA from G1163 were unsuccessful. Interestingly, sample G1588 showed close phylogenetic relatedness with Fijian collection G0311, with both samples belonging to the same subcluster in the LC-MS based HCA but exhibiting enough chemical difference to not cluster together when examined at the level of greatest detail (Figure S2, Figure 2). *Peyssonnelia* collections G0311 and G1004 appeared morphologically and chemically similar based on LC-MS and ^1^H NMR spectroscopic data, and both samples possessed high concentrations of **1**. Collectively, the close association based on LC-MS hierarchical analysis and the presence of **1** in G1163, G1588, G0311, and G1004 was complemented by analogous morphology and close phylogenetic relatedness (Figure S2). However, detailed comparison of ^1^H NMR spectroscopic data revealed additional complexity, whereby Fijian G0311 and G1004 exhibited comparable ^1^H NMR profiles, distinct from those of Solomon Island G1163 and G1588 (Figure 2). The ^1^H NMR spectroscopic data for G0311 and G1004 indicated the presence of multiple anomeric protons at 4–5 ppm, suggesting the presence of additional glycosides beyond **1** and its diglycoside peyssonnoside B (**2**).

*Peyssonnelia* collections G0581 and G0584 both exhibited promising activity against MRSA (Table 1), but they did not cluster closely based on LC-MS hierarchical analysis using negative ionization mode data for the midpolarity fraction B (Figure 2) and had substantially different ^1^H NMR spectroscopic data. Despite these differences, G0581 and G0584 showed close association via three other LC-MS based HCAs that were generated with positive and negative ionization data for the two midpolarity fractions (Figure S3). Additionally, 18S rRNA phylogenetic analysis along with morphological similarity and close proximity of these two collections supported the hypothesis that these samples were genetically related (Figure 1, Figure S2). A close inspection of key ^1^H NMR chemical shifts and comparison with existing literature data suggested the presence of sesquiterpene hydroquinones (**3**–**6**) in both samples (Figure 1, Figure S5), supported by their HRMS and MS^2^ data (data not shown).^15,16^ Hence, G0581 and G0584 reflected the bioactive sesquiterpene hydroquinone chemotype. Differences in the ^1^H NMR spectroscopic data for G0581 and G0584 were attributed to the presence of additional compounds in both G0581 and G0584 (Figure S5). Follow-up studies on the bioactive fraction led to the isolation of peyssonol A (**5**) with MIC = 3.2 μg/mL against MRSA thus validating this metabolomics approach enabling us to pinpoint the bioactive constituent from among chemically diverse but closely related group of organisms.

Additional subclusters observed in the LC-MS-based HCA were examined by overlaying their respective ^1^H NMR spectra, uncovering chemical variation present among the 32 *Peyssonnelia* collections. As illustrated in Figure 2, ^1^H NMR spectroscopic profiles for collections G1805/G1835, G1804/G1836, G1815/G1834, G0577/G0578, and G1826/G1840/G1130 were similar (within each set of two or three samples) and paralleled their relatedness as observed in LC-MS based HCA. Although some promising associations were observed between phylogeny and LC-MS based HCA for the collections, a deeper evaluation was precluded by the lack of phylogenetic data for 14 collections because of difficulties extracting and amplifying DNA from heavily encrusted specimens (Figure S2). Taken together, the LC-MS and ^1^H NMR spectroscopic data complemented each other well and, in conjunction with 18S rRNA-based phylogenetic analysis for a subset of collections, increased understanding of the chemical relatedness and diversity among the 32 *Peyssonnelia* collections.

**Figure 3:**
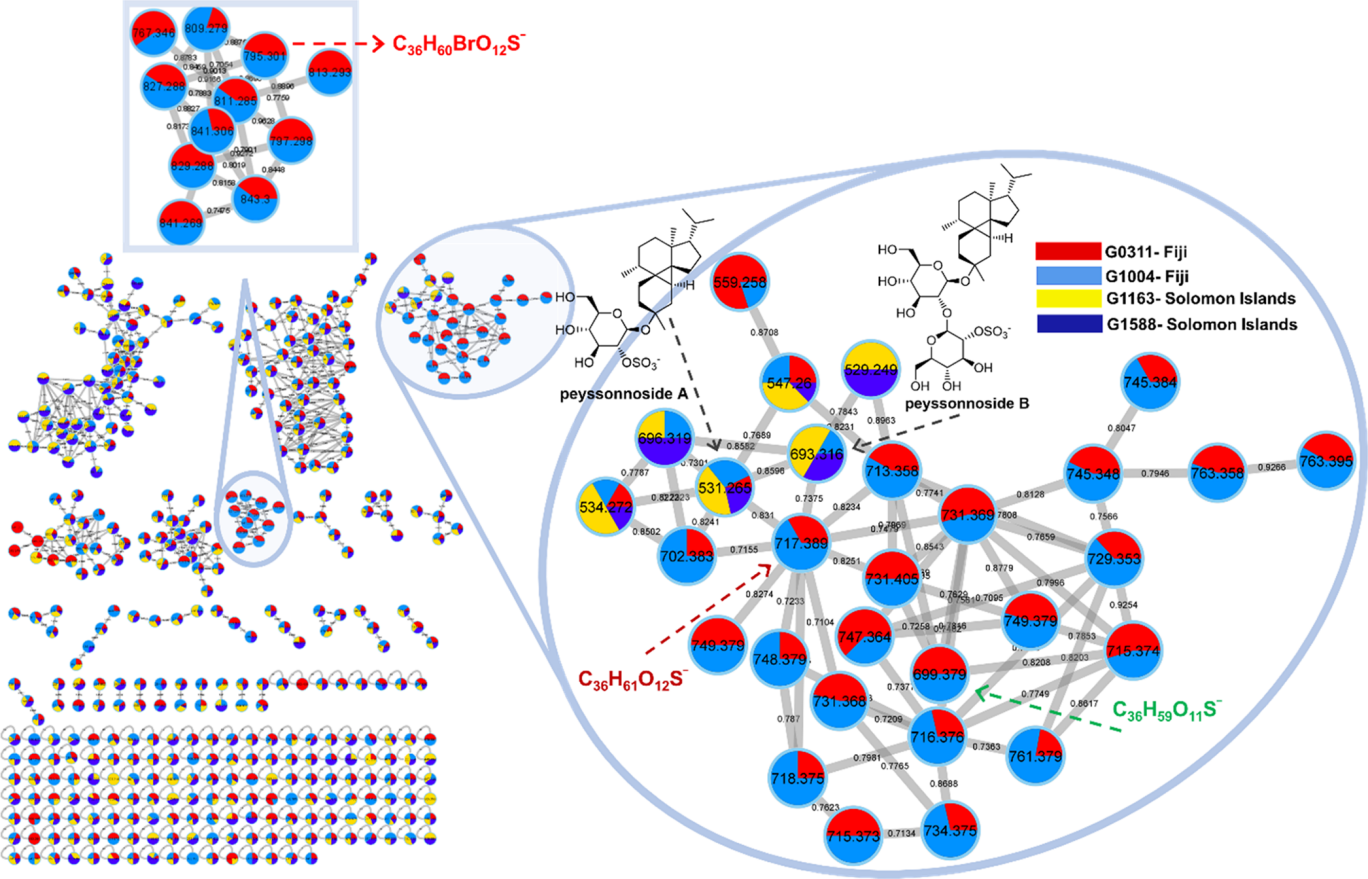
Molecular network analysis (generated using GNPS online platform and visualized using Cytoscape 3.8.0) for Fijian collections G0311, G1004 and Solomon Island collections G1163, G1588 of Peyssonnelia spp (mid polarity fractions B).^18^ Enlarged bubble depicts MS^2^ fragmentation-based molecular ion cluster (each node represents a molecular ion with color codes signifying the samples in which the molecular ions were detected) encompassing the peyssonnoside family of natural products. The Fijian samples G0311, G1004 contain novel triterpene glycosides peyssobaricanoside A–C (**15**–**17**) that exhibited similar MS^2^ fragmentation to peyssonnosides A–B (**1**–**2**).

**Figure 4:**
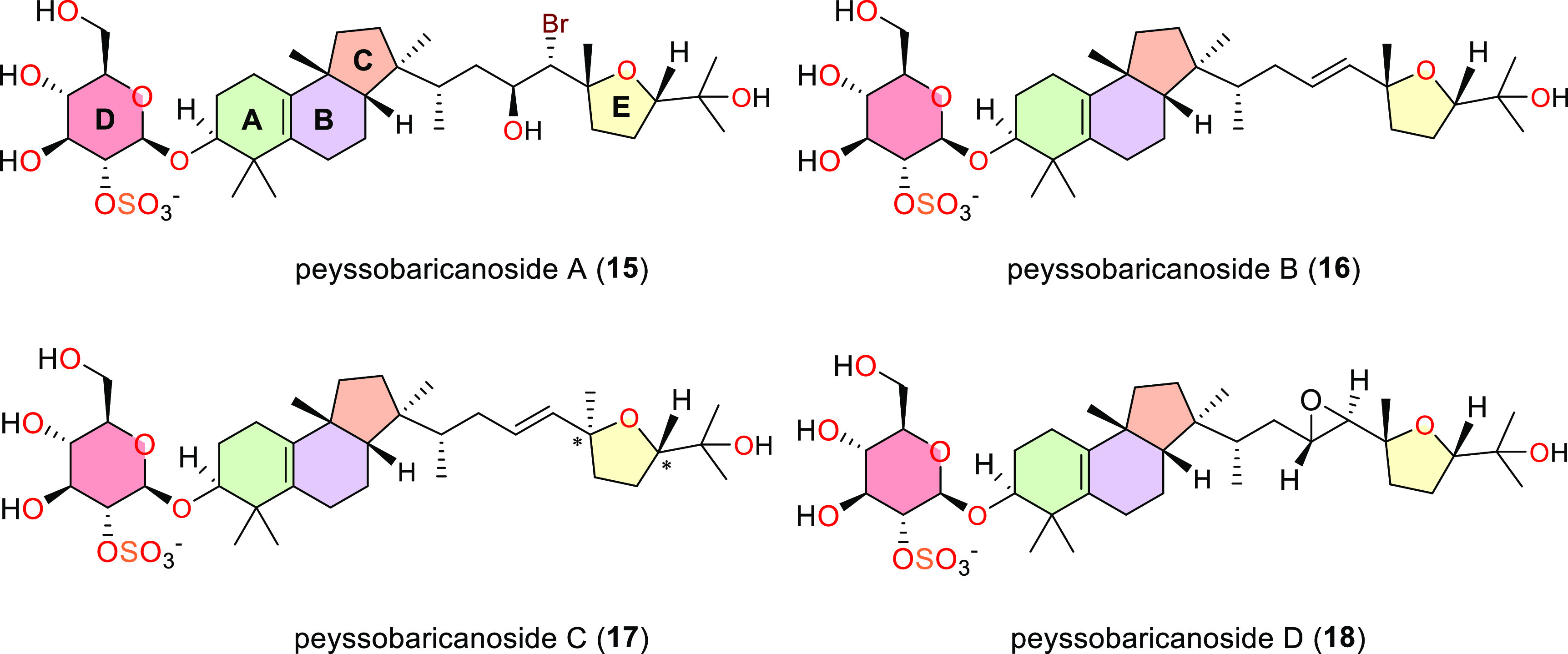
Novel triterpene glycosides peyssobaricanosides **A**–**D** (**15**–**18**) from marine red algae Peyssonnelia spp. Asterisks indicate uncertain absolute configuration for carbon positions 19 and 22, such that either 19R,22S or 19S,22R are possible for **17**.

To explore the chemical diversity of Peyssonnelia spp. in greater depth toward discovering new natural products from prioritized collections, we undertook a classical molecular network analysis on the MS^2^ fragmentation data acquired for collections G0311, G1004, G1163, and G1588 using Global Natural Product Social Molecular Networking (GNPS) (Figure 3).^17,18^ The cluster encompassing the diterpene sulfated glycoside peyssonnoside family of natural products was readily identifiable, with the monoglycosidic **1** detected in all four collections, while the less abundant diglycoside **2** was detected in three samples. Interestingly, as suggested by the molecular network data, previously unknown natural products with similar MS^2^ fragmentation to the peyssonnonsides were exclusively detected in Fijian specimens (G0311 and G1004) (Figure 3B). Comparison of MS^2^ fragmentation patterns for the molecular ion with *m*/*z* 717.389 with that of **1** (*m*/*z* 531.265) suggested a C_30_ isoprenoid aglycone coupled to a sulfated monoglycoside (Figure S4). A product ion with *m*/*z* 241 was consistent with the loss of a sulfated monoglycoside from the parent ion *m*/*z* 717.389, while a product ion at *m*/*z* 597 indicated that the molecule underwent a retro-Diels–Alder based fragmentation at the sulfated monoglycoside, leading to a neutral loss of 118 amu. A separate cluster (Figure 3C) representing molecular ions associated solely with G0311 and G1004 caught our attention, as several similar intensity ions within this cluster had a mass difference of 2 amu, suggesting the presence of brominated analogues for the C_30_ isoprenoid monoglycosides (Figure 4). Consequently, to unveil these putative C_30_ isoprenoid monoglycosides, we purified natural products with *m*/*z* 699.379 and 795.301, which we anticipated to be present at higher concentrations in G0311 based on their relative abundances in the LC-MS profiles.

**Figure 5:**
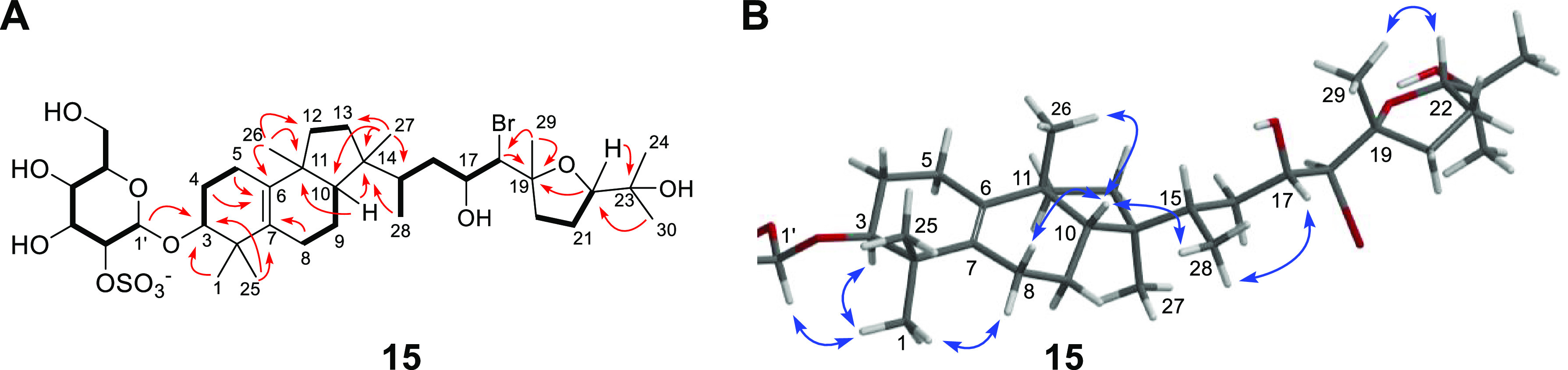
Key (A) COSY/TOCSY (bold lines), HMBC (red arrows), and (B). 1D ROESY (blue arrows) correlations for peyssobaricanoside A (**15**).

Peyssobaricanoside A (**15**), a brominated triterpene glycoside (*m*/*z* of 795.301) along with two analogues peyssobaricanoside B–C (**16**–**17**, *m*/*z* 699.379) (Figure 4), were isolated by HPLC from the midpolarity fraction (fraction B) of the Peyssonnelia sample G0311, guided by their LC-MS profiles. Based on HRAM data (*m*/*z* 795.301 [M]^−^), a molecular formula of C_36_H_60_BrO_12_S was deduced for **15**. HSQC combined with ^1^H and ^13^C NMR spectroscopic data suggested the presence of eight methyls, ten aliphatic methylenes, ten aliphatic methines, and eight quaternary centers, accounting for the 36 carbon atoms. The calculated degrees of unsaturation along with the presence of a single double bond (downfield ^13^C NMR chemical shifts of 135.3 and 136.6 ppm) indicated that the molecule contained five rings (Table 2). A sharp doublet at δ_H_ 4.51 (d, ^3^J_H-1′_, _H-2′_ = 7.7 Hz, H-1′, a β anomeric proton) and a downfield chemical shift of 104.2 ppm for the associated carbon, along with multiple proton signals at 3–5 ppm, were indicative of a monosaccharide (Table 2). Vicinal COSY correlations starting from the anomeric proton H-1′, along with 1D TOCSY data acquired by selective irradiation of H-1′ at several mixing times, revealed the H-1′/H-2′/H-3′/H-4′/H-5′/H-6′ spin system for the monosaccharide moiety (Figure 5A). Large vicinal couplings of >7 Hz were observed for all the methine protons present in the monosaccharide ring (as revealed by 1D TOCSY irradiation of H-1′, Figure S11), characteristic of a β-d-glucose moiety. The relatively downfield ^13^C chemical shift for C-2′ (δ_C_ = 81.9), compared with C-3′, C-4′, and H-5′, implied that C-2′ was sulfated, as observed with the peyssonnosides. With 11 carbon atoms to be accounted for in the downfield ^13^C region between 62–105 ppm, a sulfate moiety at C-2′ comported with the number of oxygen and sulfur atoms suggested for **15** based on the HRMS data.

**Table 2:** NMR Spectral Data for Peyssobaricanoside A (**15**) in CD_3_OD (800 MHz)

No.	*δ*_C_	*δ*_H (mult, *J* Hz)_	HMBC	COSY
1	22.1 (CH_3_)	1.01 s	C-2, C-3, C-7, C-25	
2	40.8 (C)			
3	88.9 (CH)	3.42 m	C-1, C-2, C-7, C-25, C1′	H-4a, H-4b
4a	27.5 (CH_2_)	1.72 m	C-2, C-3, C-5, C-6	H-3, H-5a, H-5b
4b		2.04 m	C-2, C-3, C-5, C-6	H-3, H-5a, H-5b
5a	25.3 (CH_2_)	1.96 m	C-3, C-4, C-6, C-7	H-4a, H-4b
5b		2.14 m	C-3, C-4, C-6, C-7	H-4a, H-4b
6	135.3 (C)			
7	136.6 (C)			
8a	25.0 (CH_2_)	1.72 m	C-2, C-6, C-7	H-9a
8b		2.13 m	C-2, C-6, C-7, C-9, C-10	H-9a, H-9b
9a	27.0 (CH_2_)	1.24 m	C-7, C-10	H-8a, H-8b, H-10
9b		1.68 m	C-7, C-8, C-10, C-11	H-8b, H-10
10	55.8 (CH)	1.51 m	C-6, C-8, C-9, C-11, C-12, C-14, C-15, C-26, C-27	H-9a, H-9b
11	47.9 (C)			
12a	36.7 (CH_2_)	1.40 m	C-10, C-11, C-26	H-13a, H-13b
12b		1.62 m	C-6, C-10, C-11, C-13, C-26	H-13a
13a	39.2 (CH_2_)	1.28 m	C-12, C-14, C-27	H-12a, H-12b
13b		1.67 m	C-10, C-11, C-12, C-14, C-15, C-27	H-12a
14	48.3 (C)			
15	42.9 (CH)	1.69 m	C-10, C-14, C-16, C-27, C-28	H-28, H-16b
16a	40.1 (CH_2_)	1.62 m	C-15, C-17, C-18, C-28	H-17
16b		1.57 m	C-15, C-17, C-18, C-28	H-15, H-17
17	71.0 (CH)	3.97 m	C-15, C-16, C-18, C-19	H-16a, H-16b, H-18
18	67.2 (CH)	4.04 d (6.7)	C-16, C-17, C-19, C-29	H-17
19	87.3 (C)			
20a	40.3 (CH_2_)	2.18 m	C-18, C-19, C-21, C-29	H-21a, H-21b
20b		1.88 m	C-18, C-19, C-21, C-22, C-29	H-21a, H-21b
21a	26.3 (CH_2_)	1.92 m	C-19, C-20, C-22, C-23	H-22, H-20a, H-20b
21b		1.92 m	C-19, C-20, C-22, C-23	H-22, H-20a, H-20b
22	87.9 (CH)	3.94 m	C-19, C-21, C-23	H-21a, H-21b
23	72.3 (C)			
24	26.6 (CH_3_)	1.2 s	C-22, C-23, C-30	
25	25.7 (CH_3_)	1.14 s	C-1, C-2, C-3, C-7	
26	29.1 (CH_3_)	1.03 s	C-6, C-10, C-11, C-12	
27	18.5 (CH_3_)	0.75 s	C-10, C-13, C-14, C-15	
28	15.2 (CH_3_)	0.94 d (6.7)	C-14, C-15, C-16	H-15
29	21.5 (CH_3_)	1.42 s	C-18, C-19, C-20	
30	25.8 (CH_3_)	1.14 s	C-22, C-23, C-24	
1′	104.2 (CH)	4.51 d (7.7)	C-3, C-3′, C-4′	H-2′
2′	81.9 (CH)	4.08 dd (7.7, 7.7)	C-1′, C-3′	H-1′, H-3′
3′	77.8 (CH)	3.68 dd (8.9, 8.9)	C-1′, C-2′, C-4′	H-2′, H-4′
4′	71.5 (CH)	3.42 dd (8.9, 8.9)	C-6′, C-4′	H-3′, H-5′
5′	77.3 (CH)	3.28 m	C-1′, C-3′, C-5′, C-6′	H-4′, H-6′a, H-6′b
6′a	62.7 (CH_2_)	3.68 m	C-4′, C-5′	H-5
6′b		3.86 dd (11.9, 2.4)	C-4′, C-5′	H-5

With four rings left to account for, the remaining 30 carbons together with eight upfield methyl peaks in the ^1^H NMR spectrum were indicative of a tetracyclic triterpene aglycone for **15** (Table 2). Starting at H-3, COSY correlations were observed between H-3/H_2_-4, H_2_-4/H_2_-5, further supported with 1D TOCSY data acquired by selective irradiation of H-3 (δ_H_ 3.42) (Figure 5A, Figure S12). Additionally, the downfield proton chemical shift for H-3 (δ_H_ 3.42) along with HMBC correlations observed from H-3 to C-1′ and H-1′ to C-3, confirmed that the sulfated monosaccharide was connected to the triterpene aglycone at C-3 (Figure 5A). HMBC correlations from H_3_-1 and H_3_-25 to C-2, C-3, and C-7 combined with key correlations observed from H_2_-5 to C-6, C-7; H_2_-4 to C-6; and H-3 to C-7 established the connectivity of ring A.

COSY correlations combined with 1D TOCSY data acquired by selective irradiation of H-10 (δ_H_ 1.51) revealed the spin systems H_2_-8/H_2_-9/H-10 and H_2_-12/H_2_-13 in **15** (Table 2, Figure S13). Together with key HMBC correlations from H_2_-8 to C-2, C-6, C-7; H_2_-9 to C-10, C-11; H-10 to C-6, C-8, C-9, C-11, C-12, C-14, C-15, C-26, C-27; H_3_-26 to C-6, C-10, C-11, C-12; H_2_-12 to C-6, C-10, C-11; H_3_-27 to C-10, C-13, C-14 established rings B and C. Interestingly, starting at H_3_-28 (d, ^3^*J*_H-28, H-15_ = 6.7 Hz) the COSY spin system H_3_-28/H-15/H_2_-16/H-17/H-18 was readily traceable. HMBC correlations observed from H_3_-27 to C-15 and H_3_-28 to C-14 clearly linked the C-15 to C-18 segment of **15** with ring C (Figure 5A). At this point, we were not confident about the regiochemistry of the bromohydrin at C-17/C-18, as C-17 and C-18 had comparable carbon chemical shifts, δ_C_ of 71.0 and 67.2 respectively, and thus required additional experiments for an unambiguous assertion (Table 2).

With one additional ring to be placed and eight carbon atoms remaining (three methyls, two quaternary centers, two methylenes, and one methine), the downfield carbon shifts observed for C-19 (δ_C_ 87.3) and C-22 (δ_C_ 87.9) were suggestive of a tetrahydrofuran ring in **15**. The ^1^H–^1^H spin system H_2_-20/H_2_-21/H-22 was delineated from the COSY spectrum, supported with 1D TOCSY data acquired by selective irradiation of H-22 (δ_H_ 3.94, Figure S14). While HMBC correlations from H_2_-20 to C-19, H-22 to C-19 and from H_3_-29 to C-20 further supported the presence of a tetrahydrofuran ring, correlations observed from H_3_-29 to C-18 and C-19 along with correlations from H-18 to C-19 and C-29 connected the tetrahydrofuran ring to the acyclic segment of the aglycone. Finally, HMBC correlations from H-24, H-30 to C-22 and H-22 to C-23 revealed the position of the propan-2-ol moiety (Figure 5, Table 2).

Returning to the ambiguous regiochemistry of the bromohydrin moiety at C-17/C-18, we conducted a hydrogen/deuterium exchange experiment, predicting that the chemical shift difference (comparison of ^13^C chemical shift acquired for **15** in CD_3_OD and CD_3_OH) for the carbon atom bearing a bromine would be small as compared with the carbon that positioned a hydroxyl of the bromohydrin moiety. Unfortunately, results were inconclusive as both C-17 and C-18 showed large Δ δ_C_ values of 24 and 25 Hz, respectively (Figure S15). Our second approach was to leverage isotopic shifts induced in a ^13^C NMR spectrum by ^79^Br and ^81^Br. However, this experiment turned futile as signal-to-noise ratios for C-17 and C-18 were not optimal for resolution enhancement using Gaussian window functions, despite the acquisition of a large number of scans (data not shown). Finally, the regiochemistry of the bromohydrin moiety was unambiguously assigned by comparing the ^13^C NMR chemical shifts of the bromohydrin moiety with synthesized model compounds and similar structural motifs that were reported in the literature (Figure S16).^19−21^

**Figure 6:**
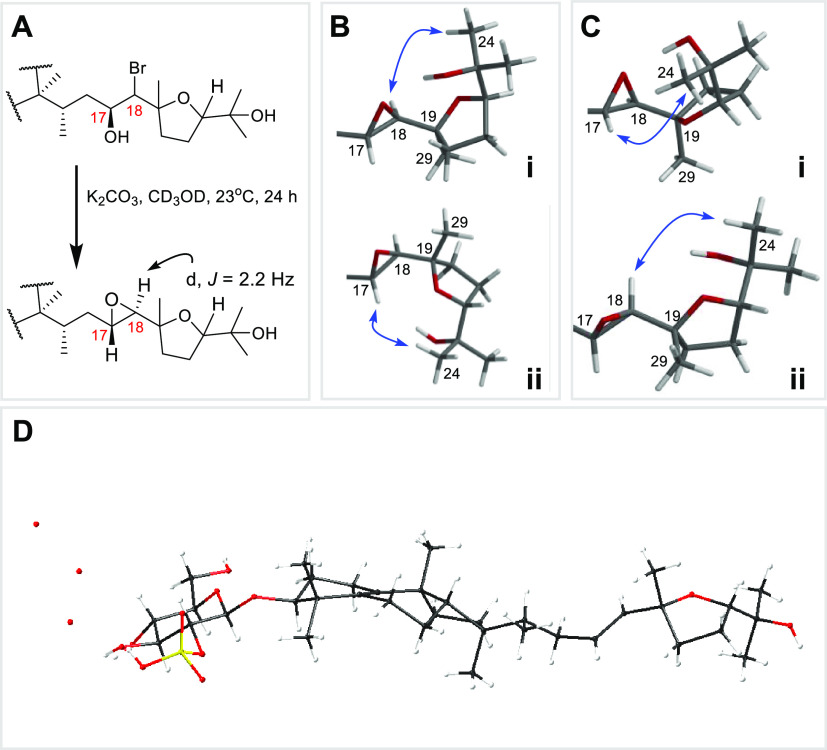
(A) Conversion of halohydrin moiety present in peyssobaricanoside A (**15**) into an epoxide to aid with stereochemical analysis. (B, C) Experimental 1D ROESY correlations (blue arrows) observed for **15** are satisfied by both 19R,22S (B i, ii) and 19S,22R (C i, ii). Additionally, the unusually large experimental ^3^*J*_CH_ observed between H-18 and C-19 (^3^*J*_H-18, C-19_ = 8.0 Hz) was also satisfied by the computed *J* values for both models B i, ii and C i, ii (for computational details refer Figure S36). (D). MicroED structure of **16** illustrating configuration of the tetrahydrofuran moiety.

Moving forward to the determination of relative and absolute configuration, 1D selective ROESY correlations observed between H_3_-26 and H-10 established a *cis* relative configuration between rings B and C of **15** (Figure 5B). ROESY correlations were also observed between H-10/H_3_-28 and H_3_-27/H_3_-28 thus establishing the relative configuration of H_3_-28 with respect to ring C. 1D ROESY correlations were observed between H_3_-28/H17 and H_3_-28/H-18, suggesting that the C15–C19 fragment of the molecule was flexible. Interestingly, acquisition of ^1^H NMR data in a mixture of CD_3_CN/DMSO-*d*_6_ (1:3) showed that H-16a and H-16b appeared as distinct doublet of doublets with large coupling constants (dd, ^3^*J*_H-16a, H-15_ = 12.1 Hz, ^3^*J*_H-16a, H-16b_ = 12.1 Hz for H-16a and dd, ^3^*J*_H-16b, H-17_ = 12.2 Hz, ^3^*J*_H-16b, H-16a_ = 12.2 Hz for H-16b, Figure S22). Additionally, a strong 1D ROESY correlation was observed between H_3_-28/H-17 but not for H_3_-28/H-18, indicative that the flexibility of C15–C19 was comparatively restricted when **15** was dissolved in CD_3_OD/DMSO-*d*_6_ (1:3) (Figure S23). Hence, the molecular fragment C-28/C-15/C-16/C-17 assumed a chair conformation, uncovering the relative configuration between H_3_-28 and H-17. Determining the relative configuration at C-18 (δ_H_ 4.04 d, ^3^*J*_H-18, H-17_ = 6.7 Hz, in CD_3_OD) turned out to be challenging as C-17/C-18 bond rotation precluded *J*-based configurational analysis. Gratifyingly, treatment of **15** with K_2_CO_3_ furnished the *trans*-epoxide peyssobaricanoside D (**18**, H-18, δ_H_ 4.04 d, ^3^*J*_H-18, H-17_ = 2.2 Hz, NOE was not observed between H-17 and H-18, Figure 6A). While the relative configuration of the tetrahydrofuran ring was established by 1D ROESY correlations observed between H_3_-29 and H-22, the absolute configuration at C-19 and C-22 could not be determined based on ROESY, experimental and DFT based *J* coupling analysis. The unusually large experimental ^3^*J*_CH_ observed between H-18 and C-19 (^3^*J*_H-18, C-19_ = 8.0 Hz) was satisfied by the computed *J* values for both models. Both stereoisomers in consideration were predicted by DFT calculations to have *J*_C–H_ values of 8 Hz (Figure 6). Delightfully, although severely sample-limited, **16** generated microcrystals which were subjected to cryogenic electron microscopy (cryo-EM) based microcrystal electron diffraction (MicroED) and hence resolved the absolute configuration (Figure 6D). Consequently, we propose the absolute configuration of **15** as 1′*R*,2′*R*,3′*S*,4′*S*,5′*R*,3*S*,10*S*,11*R*,14*S*,15*S*,17*S*,18*S*,19*R*,22*S* presuming that **15** is a halohydrin product of **16**. While ^1^H and ^13^C NMR spectroscopic data for **17** paralleled that of **15**–**16** and 1D ROESY NMR data suggested that **17** had a *trans*-relative configuration between H_3_-29/H-22, the absolute configuration at C-29 and C-22 for **17** remains uncertain (Figure 4).

**Figure 7:**
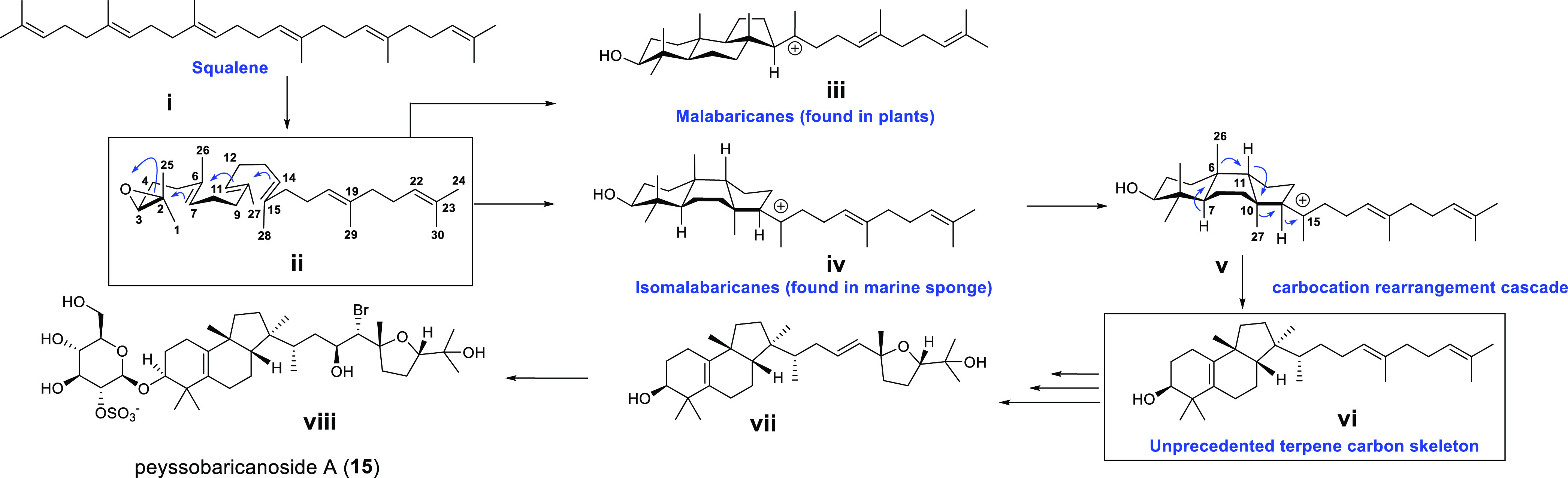
Proposed biosynthetic scheme for peyssobaricanoside A (**15**) starting from squalene.

From a biosynthetic perspective, we envision that the initial squalene cyclization for building the tricyclic aglycone core (rings A, B, and C) of peyssobaricanosides is analogous to the biosynthesis of isomalabaricane carbon skeleton observed in natural products from marine sponges (Figure 7, **i**, **ii**, and **iv**). However, in marine algae of the genus *Peyssonnelia*, the resulting biosynthetic intermediate **iv** is postulated to undergo a carbocation rearrangement cascade resulting in a novel carbon skeleton **vi**. Subsequent steps of epoxidation and ring cyclization **viii**, halohydrin formation **x**, and glycosylation forms peyssobaricanoside A (**15**).

## Experimental Procedures

### General Experimental Procedures

Fractionation of algal extracts were performed with vacuum liquid chromatography (VLC) using Diaion HP-20SS resin styrenic adsorbent (particle size: 75–150 μm, porosity: 520 Å) as stationary phase. High-performance liquid chromatography (HPLC) separations were achieved either using Waters 2695 separation module equipped with a Waters Acquity QDa mass detector or Waters 1525 binary pump monitored with an Altech ELSD 800 detector. The HPLC separations were monitored using Waters MassLynx 4.1 or Waters Empower 3 software. NMR spectroscopic data (^1^H, ^13^C, 1D TOCSY, 1D ROESY, COSY, HSQC, and HMBC) were acquired on 18.8 T Bruker Advance IIIHD instrument (800 MHz for ^1^H and 200 MHz for ^13^C) equipped with a 3 mm triple resonance broadband cryoprobe. Spectra were recorded in deuterated solvents and referenced to the solvent residual peaks (δ_H_ 2.50, δ_C_ 39.52 for DMSO-*d*_6_, δ_H_ 3.31, δ_C_ 49.00 CD_3_OD, and δ_H_ 7.26, δ_C_ 77.16 for CDCl_3_). NMR spectroscopic data were processed and analyzed using MestReNova 11.0.4.

### Specimen Collection and Identification

Collection details (year, location, GPS coordinates, abundance, and morphology) for the 32 *Peyssonnelia* spp. are reported in Table S1. Photos generated during field collection or taken for algal samples in the lab (after thawing collections preserved at −80 °C) is provided in Figure 1 and Figure S1. Morphological voucher samples were preserved in formalin and DNA vouchers were stored in molecular grade ethanol at Georgia Tech.

The *Peyssonnelia* spp. were identified by morphological features with that of previously described *Peyssonnelia* species and by nuclear, small subunit (SSU) rRNA (18S rRNA) sequence analysis.^22^ Genomic DNA from ethanol-preserved algal specimen were extracted using the innuPREP plant DNA kit (Analytik Jena, Germany) according to the manufacturer’s protocol. From each species’ genomic DNA, four overlapping 18S rRNA gene fragments were amplified via the polymerase chain reaction (PCR) in four separate reactions using (G01/G10, G02/G14, G04/G13, and G06/G07) primers.^23^ Each PCR amplification was performed in a 25 μL reaction volume consisting of 5–50 ng of purified genomic DNA; 200 μM of each of the dNTPs; 1 μM of each of the oligonucleotide primer and 1.0 U Taq DNA Polymerase; and 1× Standard PCR reaction buffer (NEB, Ipswich, MA). All PCR amplifications were performed in a GeneAmp PCR system 2700 (Applied Biosystems, Foster City, CA) thermocycler using the following temperature cycling parameters: initial denaturation at 94 °C for 5 min followed by a total of 40 cycles of amplification in which each cycle consisted of denaturation at 94 °C for 40 s, primer annealing at 50 °C for 40 s, and primer extension at 72 °C for 1 min. After amplification, final extension of the incompletely synthesized DNA was carried out at 72 °C for 7 min. The PCR fragments were analyzed by agarose gel electrophoresis (1 wt %/vol). The gel was stained with ethidium bromide and visualized under a UV transilluminator. All the PCR fragments were either sequenced with forward and reverse primers, and sequences were manually edited and assembled using CAP3 Sequence Assembly Program.^24^ The assembled *Peyssonnelia* spp. 18S rRNA sequences were submitted to GenBank (accession no: OP032191–OP032209, and MK129455). The sequence similarity of the assembled contig of *Peyssonnelia* spp. 18S rRNA to other known red algae from family Peyssonneliaceae was determined by comparing it with the nonredundant nucleotide database (NCBI) using the blastn program.^25^ Based on E-values and maximum scores, all the sequenced species matched to multiple species within the order Gigartinales. To place these *Pyssonnelia* spp. phylogenetically within the Family Peyssonneliaceae (Order Gigartinales), the V4 region of their 18S rRNA sequences were compared with those of known representatives from Family Peyssonneliaceae, obtained from GenBank. Phylogenetic analysis was conducted in MEGA X using the Maximum Likelihood method based on the Kimura 2-parameter model with 1000 bootstrap iterations (Figure S2).^26,27^

### Extraction, Fractionation of Algal Samples, and Isolation of Peyssobaricanoside A*–*C (**15**–**17**)

Each algal collection (wet sample, 32 in total) was extracted with methanol (three times) and subjected to VLC fractionation using HP20SS Diaion resin (ratio of dry extract to resin was 1:20). Four fractions were generated by eluting with 50% aqueous methanol (A), 80% aqueous methanol (B), methanol (C), and then acetone (D). Fractions A and D were not subjected to NMR or HRMS based metabolomics or bioassay as they primarily contained salts, lipids, and algal pigments.

*Peyssonnelia* sp. (G0311, 74 g wet weight) was extracted with 80% aqueous methanol, 100% methanol, and 1:1 methanol/dichloromethane yielding 1.7 g of crude extract. HP20SS Diaion resin (40 g) was used to adsorb the crude extract (1.7 g), washed with water to remove salts, and then eluted with 50% aqueous methanol (A), 80% aqueous methanol (B), methanol (C), and acetone (D) to generate four fractions A (0.80 g), B (0.13 g), C (0.25 g), D (0.059 g), and water-soluble fraction (0.47 g). Fraction B was subjected to preparative silica TLC (Silicycle 200 μm, 20 × 20 cm) using 1:55:200 ratio of water (0.05% TFA)/methanol/chloroform to furnish five fractions (B.1*–*B.5). A portion (19 mg) of B.2 (27 mg, *R*_f_ 0.26) was further separated in a reversed phase HPLC column (Alltima C18, 5 μm, 4.6 × 250 mm) using the solvent gradient as shown in Table S2. While fractions eluting between 9*–*11 min contained a mixture of **16** and **17**, fractions between 16.5*–*18.5 min contained partially pure **15**, which was characterized using NMR and mass spectrometry without further purification. The fractions eluting between 9*–*11 min (∼2.0 mg) were subjected to reversed phase HPLC separation (ZORBAX SB-C18, 5 μm, 4.6 × 250 mm) using isocratic isocratic 88:12 ratio of 2:8 acetonitrile/water and isopropanol eluting **16** (0.2 mg) and **17** (0.2 mg) at 10.4 and 11.1 min, respectively.

### Derivatization of **15** to **18**

A portion of partially pure **15** was dissolved in methanol, mixed with K_2_CO_3_ (0.4 mg), and stirred at room temperature for 2 h. The reaction mixture was neutralized and subjected to reversed phase HPLC column (Alltima C18, 5 μm, 4.6 × 250 mm) using a solvent gradient between 2:8 acetonitrile/water and isopropanol, starting at a ratio of 8:2 to 1:1 over 12 min, eluting **18** (0.1 mg) at 7 min.

### Synthesis of Model Compound 2-(5-(1-Bromo-2-hydroxyethyl)-5-methyltetrahydrofuran-2-yl) Propan-2-ol

Refer to figure legend Figure S16

### Computational Procedure

A section of the proposed structure for peyssobaricanoside D (**18**) as represented by **a**–**f** (models generated using Spartan Student v.7.2.7, Figure S36) were subjected to DFT based structural optimization and total nuclear spin–spin coupling *J* (Hz) prediction using B3LYP/6-31G(d,p) as implemented in Gaussian 16 software.^28,29^

### Antimicrobial Assays

Antibacterial and antifungal screening were conducted as reported earlier, with the MIC being defined as the minimum concentration required to achieve ≥90% inhibition of optical density (OD_600 nm_)in comparison to the vehicle control.^30^ HP20SS fractions B and C were tested against *Escherichia coli* (EC, ATCC 25922), multidrug-resistant *E*. *coli* (MDREC, ATCC BAA-1743), methicillin-resistant *Staphylococcus aureus* (MRSA, ATCC 33591), vancomycin-resistant *Enterococcus faecium* (VREF, ATCC 700221), and amphotericin B-resistant *C*. *albicans* (ARCA, ATCC 90873). Chloramphenicol was used as the positive control for EC and VREF, nitrofurantoin for MDREC, vancomycin for MRSA, and cycloheximide for ARCA. Additional antimicrobial assays were conducted against *Enterobacter cloacae* (CDC0008), *Klebsiella pneumoniae* (CDC0016), *Acinetobacter baumannii* (CDC0033), and the PAO1 strain of *Pseudomonas aeruginosa* (PAO1). Bacteria were maintained on tryptic soy agar (TSA) plates and overnight cultures were grown in tryptic soy broth (TSB). Bacterial cultures for experiments were grown in cation-adjusted Mueller Hinton broth (CAMHB) following CLSI guidelines.^31^ Bacteria were grown at 35 °C in a humidified growth chamber. The *P*. *aeruginosa* strain used in these experiments were provided by Dr. Alex Horswill (University of Colorado). All other strains were acquired from the CDC & FDA Antibiotic Resistance (AR) Isolate Bank. Microbroth dilution in a 384-well plate was used to determine bacterial growth inhibition, according to CLSI guidelines.^31^ Bacterial overnight cultures were grown in tryptic soy broth (TSB) and then standardized to 5 × 10^5^ CFU/mL in CAMHB for experimental cultures. Extracts were diluted in CAMHB in triplicate at a concentration of 125 μg/mL with a final well volume of 30 μL via a Liquid Handling Station (BrandTech), and absorbance of wells were measured at 600 nm with a BioTek Cytation3 plate reader before and after incubation (18 h). Growth inhibition was measured relative to vehicle control (DMSO). Positive controls used were tetracycline for *A*. *baumannii* and Meropenem for *E*. *cloacae*, *K*. *pneumoniae*, and *P*. *aeruginosa*. A media blank and growth control were included for each experiment, and each experiment was performed twice on separate days. Wells contained either 5% or 10% (for two out of 64 extract fractions assayed) DMSO. Besides the MRSA results shown in Table 1, extract fractions did not show significant bioactivity against tested pathogens.

### Antimalarial Assay

Liver-stage antimalarial screening against *Plasmodium berghei* was conducted as reported earlier.^32^

### Mass Spectrometry-Based Metabolomics Study

Dried aliquots of fraction B (eluting with 80% methanol in HP20SS Diaion resin) and fraction C (eluting with 100% methanol in HP20SS Diaion resin) for each algal collection (32 in total) were each reconstituted in MeOH (containing 12 internal standards) to make a 1 mg/mL sample and analyzed with ultraperformance liquid chromatography (UPLC)-MS methods to collect metabolomic profiles. Internal standards were obtained from Avanti Polar Lipids, Inc., including the following, listed with their final target concentration diluted in Optima chloroform (Thermo Fisher): 160 μg/mL 15:0–18:1(d7); 5 μg/mL 15:0–18:1(d7) PE; 5 μg/mL 15:0–18:1(d7) PS; 30 μg/mL 15:0–18:1(d7) PG; 10 μg/mL 15:0–18:1(d7) PI; 25 μg/mL 18:1(d7) LPC; 5 μg/mL 18:1(d7) LPE; 350 μg/mL 18:1(d7) Chol Ester; 10 μg/mL 15:0–18:1(d7) DG; 55 μg/mL 15:0–18:1(d7)-15:0 TG; 30 μg/mL 18:1(d9) SM; 100 μg/mL Cholesterol (d7). Sample blanks were created by eluting HP20SS Diaion resin with 80% methanol (blank for fraction B) and then with 100% methanol (blank for fraction C). For quality control purposes, a pooled sample was created by mixing an equal volume from each sample extract.

UPLC-MS analysis used a Vanquish (Thermo Fisher Scientific), fitted with a Thermo Fisher Scientific Accucore C30 column (2.1 × 150 mm, 2.6 μm particle size), coupled to a high-resolution accurate mass Orbitrap ID-X mass spectrometer system (Thermo Fisher Scientific). The chromatographic method for sample analysis involved elution with 80:20 water:acetonitrile with 10 mM ammonium formate and 0.1% formic acid (mobile phase A) and 10:90 acetonitrile:isopropyl alcohol, with 10 mM ammonium formate and 0.1% formic acid (mobile phase B) using the following gradient program: 0 min 95% A; 0.5 min 95% A; 9 min 0% A; 10.9 min 0% A; 11 min 95% A; and held until 12 min. The flow rate was set at 0.40 mL/min. The column temperature was set to 50 °C, and the injection volume was 2 μL.

The Orbitrap ID-X is a tribrid spectrometer that utilizes quadrupole isolation with dual detectors, an orbitrap, and an ion trap, with a maximum resolving power of 500,000 fwhm at *m*/*z* 200 and mass accuracy of <1 ppm. The heated electrospray ionization (HESI) source was operated at a vaporizer temperature of 275 °C, a spray voltage of 3.5 kV(+)/2.5 kV(−), and sheath, auxiliary, and sweep gas flows of 40, 8, and 1, respectively. The instrument acquired full MS data between 150 and 2000 *m*/*z* in positive or negative ionization mode. UPLC-MS/MS experiments were performed by acquiring mass spectra in a data dependent acquisition fashion. Full MS spectra were collected with a resolution of 120,000, while the dd-MS^2^ were isolated with a 0.4 *m/*z window and collected at a resolution of 30,000 with a cycle time of 1.5 s. Precursors were activated by CID with a normalized collision energy of 30%. Dynamic exclusion was set at 8 s. An exclusion list was generated from the sample blank to avoid selection of background ions during MS/MS collection.

A UPLC-MS feature list was generated following data acquisition and processing with Compound Discoverer V3.0 (Thermo Fisher Scientific). The Dataset was annotated by MS^2^ spectral matching to a local spectral database, built from curated experimental data. In addition, accurate mass, retention time, and isotopic pattern were used to match with database entries. Molecular networks for MS^2^ data were generated using the Global Natural Products Social Molecular Networking (GNPS) online platform (www.gnps.ucsd.edu) and visualized using Cytoscape 3.8.0 (www.cytoscape.org).^18^ The data was filtered by removing all MS/MS fragment ions within ±17 Da of the precursor *m*/*z*. MS/MS spectra were window filtered by choosing only the top 6 fragment ions in the ±50 Da window throughout the spectrum. The precursor ion mass tolerance was set to 0.02 Da and a MS/MS fragment ion tolerance of 0.02 Da. A network was then created where edges were filtered to have a cosine score above 0.7 and more than 4 matched peaks. Further, edges between two nodes were kept in the network if and only if each of the nodes appeared in each other’s respective top 10 most similar nodes. Finally, the maximum size of a molecular family was set to 100, and the lowest scoring edges were removed from molecular families until the molecular family size was below this threshold. The spectra in the network were then searched against GNPS spectral libraries. The library spectra were filtered in the same manner as the input data. All matches kept between network spectra and library spectra were required to have a score above 0.7 and at least 6 matched peaks. The UPLC-MS data set was centered, scaled, and subjected to Ward algorithm (Euclidean distance was used) based Hierarchical cluster analysis (HCA) in RStudio (version 3.6.0).^33^

### MicroED Methodology and Data

See SI.

## Supporting Information Available

Additional sample details, images, and spectra (PDF)
