# Supplementary material for: Cryptic Chemical Variation in a Marine Red Alga as Revealed by Nontargeted Metabolomics
Source: ACS Omega. 2023 Apr 6;8(15):13899–910. doi: 10.1021/acsomega.3c00301 (PMC10116502; doi:10.1021/acsomega.3c00301)
Supplement: Supplementary file 1 — ao3c00301_si_001.pdf [file ao3c00301_si_001.pdf]

Cryptic chemical variation in a marine red alga as revealed by non-targeted metabolomics

Bhuwan Khatri Chhetri,[†,‡] Nazia Mojib,[@] Samuel G. Moore,[Θ] David A. Delgadillo,[#] Jessica E. Burch,[#] Nolan H. Barrett,[δ] David A. Gaul,[†, Θ] Lewis Marquez,[∇] Katy Soapi,[ξ] Cassandra L. Quave,[∇] Hosea M. Nelson,[#] and Julia Kubanek[*, †, ‡,§,Θ]

[†] School of Chemistry and Biochemistry, Georgia Institute of Technology, Atlanta, GA 30332, USA

[‡] Center for Microbial Dynamics and Infection, Georgia Institute of Technology, Atlanta, GA 30332, USA

[§] School of Biological Sciences, Georgia Institute of Technology, Atlanta, GA 30332, USA

[@] Department of Biology, Spelman College, Atlanta, GA 30314, USA

[∇] Department of Dermatology, Center for the Study of Human Health, and Antibiotic Resistance Center, Emory University, Atlanta, Georgia 30322, United States

[ξ] Institute of Applied Sciences, University of South Pacific, Suva, Fiji

[Θ] Parker H. Petit Institute for Bioengineering and Bioscience, Georgia Institute of Technology, Atlanta, GA 30332, USA

[δ] School of Earth and Atmospheric Sciences, Atlanta, GA 30332, USA

[#]Division of Chemistry and Chemical Engineering, California Institute of Technology, Pasadena, California 91125, United States

Content

Table S1	Collection details for the 32 *Peyssonnelia* spp. samples	S3
Figure S1	Images for Fijian and Solomon Island collections	S5
Figure S2	Evolutionary relationship of 18 *Peyssonnelia* spp. samples and correlations observed between phylogeny and hierarchical cluster analysis of LC-MS data	S6
Figure S3	Hierarchical cluster analysis for HRMS data	S7
Figure S4	Comparison of MS^2 fragmentation pattern of peyssobaricanosides B-C with peyssonnoside B	S8
Figure S5	Overlay of ^{1}H NMR spectroscopic data for mid-polarity HP20SS fraction B of G0584 and G0581	S9
Table S2	Solvent gradient used for HPLC separation of G0311	S9
Figure S6	^{1}H NMR spectrum for peyssobaricanoside A (**15**)	S10
Figure S7	^{13}C NMR spectrum for peyssobaricanoside A (**15**)	S10
Figure S8	HSQC NMR spectrum for peyssobaricanoside A (**15**)	S11
Figure S9	COSY NMR spectrum for peyssobaricanoside A (**15**)	S11
Figure S10	HMBC NMR spectrum for peyssobaricanoside A (**15**)	S12
Figure S11	1D TOCSY (H-1′ irradiated) NMR spectrum for peyssobaricanoside A (**15**)	S12
Figure S12	1D TOCSY (H-3 irradiated) NMR spectrum for peyssobaricanoside A (**15**)	S13
Figure S13	1D TOCSY (H-10 irradiated) NMR spectrum for peyssobaricanoside A (**15**)	S13
Figure S14	1D TOCSY (H-22 irradiated) NMR spectrum for peyssobaricanoside A (**15**)	S14
Figure S15	Solvent isotope effect for ^{13}C NMR chemical shifts for peyssobaricanoside A (**15**)	S14
Figure S16	^{13}C NMR chemical shifts for hydroxyl-containing carbons adjacent to tetrahydrofuran ring: literature values, synthetic scheme, and NMR data	S15
Figure S17	^{1}H NMR spectrum for reaction of linalool-oxide with NBS	S16
Figure S18	^{13}C NMR spectrum for reaction of linalool-oxide with NBS	S16
Figure S19	1D ROESY (H-10 irradiated) NMR spectrum for peyssobaricanoside A (**15**)	S17
Figure S20	1D ROESY (H_3-28 irradiated) NMR spectrum for peyssobaricanoside A (**15**) in CD_3OD	S17
Figure S21	1D TOCSY (H_3-28 irradiated) NMR spectrum for peyssobaricanoside A (**15**) in CD_3OD:DMSO-d_6	S18
Figure S22	1D TOCSY (H_3-28 irradiated) NMR spectrum for peyssobaricanoside A (**15**) in CD_3OD:DMSO-d_6 expanded region	S18

Figure S23	1D ROESY (H_3-28 irradiated) NMR spectrum for peyssobaricanoside A (**15**) in CD_3OD:DMSO-d_6 expanded region 2	S19
Figure S24	1D ROESY (H-29 irradiated) NMR spectrum for peyssobaricanoside A (**15**)	S19
Figure S25	^1H NMR spectrum for peyssobaricanoside B (**16**)	S20
Figure S26	^{13}C NMR spectrum for peyssobaricanoside B (**16**)	S20
Figure S27	1D ROESY NMR spectrum for peyssobaricanoside B (**16**)	S21
Figure S28	^1H NMR spectrum for peyssobaricanoside C (**17**)	S21
Figure S29	^{13}C NMR spectrum for peyssobaricanoside C (**17**)	S22
Figure S30	1D ROESY NMR spectrum for peyssobaricanoside C (**17**)	S22
Figure S31	^1H NMR spectrum for peyssobaricanoside D (**18**)	S23
Figure S32	^{13}C NMR spectrum for peyssobaricanoside D (**18**)	S23
Figure S33	1D ROESY NMR spectrum for peyssobaricanoside D (**18**)	S24
Figure S34	HMBCCETGPJCL2ND NMR spectrum for peyssobaricanoside D (**18**) (expanded region 1)	S24
Figure S35	HMBCCETGPJCL2ND NMR spectrum for peyssobaricanoside D (**18**) (expanded region 2)	S25
Figure S36	Viable conformations of the epoxide in peyssobaricanoside D (**18**)	S26
Table S3	Optimized atomic coordinates for conformation a	S27
Table S4	Optimized atomic coordinates for conformation b	S28
Table S5	Optimized atomic coordinates for conformation c	S28
Table S6	Optimized atomic coordinates for conformation d	S29
Table S7	Optimized atomic coordinates for conformation e	S30
Table S8	Optimized atomic coordinates for conformation f	S30
MicroED Sample Preparation		S31
Figure S37	MicroED structure of peyssobaricanoside B (**16**)	S32
MicroED data collection		S33
MicroED structure solution and refinement		S33
MicroED special refinement details		S34
References		S34

Table S1. Collection details for the 32 *Peyssonnelia* spp. samples

ID	Year	Location	GPS	Depth	Abundance	
**G0088**	**2006**	Waitabu, Taveuni Island, Fiji	16° 48′ 58.3″ S<br>179° 50′ 49.2″ W	15–25 m, reef slope	common	maroon/red color, hard and calcified texture, fan shaped, about 2–25% epibiont coverage.
**G0214**	**2007**	Coral Coast, Viti Levu, Fiji	18° 12′ 06.5″ S<br>177° 39′ 7.2″ E	8–15 m,<br>underside of ledges	abundant	maroon crust, growing under ledges in large masses, leafy paper like texture.
**G0311**	**2007**	Lau, Vanua Balavu Island, Fiji	17° 13′ 43.3″ S<br>179° 00′ 50.4″ W	20 m,<br>reef slope	rare	red/orange color, hard and encrusting, fan shaped, about 0–1 % epibiont coverage.
**G0407**	**2007**	Lau, Vanua Balavu Island, Fiji	17° 10′ 59.5″ S<br>178° 43′ 19.2″ W	10–20 m,<br>reef slope	common	red color, leafy, slight calcification
**G0408**	**2007**	Lau, Vanua Balavu Island, Fiji	17° 10′ 59.5″ S<br>178° 43′ 19.2″ W	10–20 m,<br>reef slope	common<br>rare	maroon color, encrusting, potato chip like, flattened, fan shaped, about 0–1 % epibiont coverage.
**G0565**	**2008**	Waya, Fiji	17° 16′ 24.2″ S<br>177° 06′ 03.6″ E	20 m	ND	red color, slightly encrusting, leafy, leather, malleable.
**G0577**	**2008**	Kadavu Island, Fiji	19° 05′ 13.9″ S<br>178° 14′ 09.6″ E	20–30 m	common	dark red/wine color, blades 2–8 cm wide and 1–2 mm thick, growing on surfaces of rocks, no variegation, lobes didn’t lie flat, medium hard texture, calcified.
**G0578**	**2008**	Kadavu Island, Fiji	19° 05′ 13.9″ S<br>178° 14′ 09.6″ E	subtidal	common	dark pink/wine color, blades 2–8 cm wide and 1–2 mm thick, orange margin around blade, encrusted, calcified, potato chip like texture, beige underside, growing on rocks.
**G0581**	**2008**	Kadavu Island, Fiji	19° 03′ 04.0″ S<br>178° 25′ 44.4″ E	12 m<br>overerhangs	common	red/marron grey color, warty like surface with irregular pumps, leathery texture.
**G0584**	**2008**	Kadavu Island, Fiji	19° 02′ 32″ S<br>178° 26′ 54″ E	15 m<br><br>underside of ledges	rare	red/maroon crust with bright mustard-colored streaks, very irregular surface, 1–10 cm wide blades and 1 mm thick, beige underside, leathery in texture.
**G0801**	**2010**	Makuluva Island, Fiji	18° 11′ 12″ S<br>178° 31′ 34″ E	10 m	abundant	red color, hard and calcified texture.
**G0986**	**2011**	Nadroga-Navosa, Fiji	18° 12′ 52″ S<br>177° 42′ 40″ E	9–12 m	abundant	red colored surface with light brown underside, had symbionts including small hydroids and nudibranch, leaf like, brittle, calcareous texture.
**G1004**	**2011**	Ogea Island, Fiji	19° 10′ 48″ S<br>178° 27′ 36″ W	5–7 m	common	maroon colored surface with white underside, hard and calcified texture, brittle.
**G1130**	**2012**	Tetepare, Solomon Islands	8° 45′ 00.0″ S<br>157° 28′ 12.0″ E	16 m	common	maroon colored surface, leathery and pliable texture.
**G1134**	**2012**	Tetepare, Solomon Islands	8° 45′ 00.0″ S<br>157° 28′ 12.0″ E	16 m<br>under overhangs	common	red colored surface, crustose, coralline, and crunchy texture.
**G1163**	**2012**	Tetepare, Solomon Islands	8° 42′ 36.0″ S<br>157° 26′ 24.0″ E	3–5 m	common	red colored surface, hard, crustose, crunchy, and brittle texture.
**G1205**	**2012**	Naru, Gizo Island, Solomon Islands	8° 06′ 00.0″ S<br>156° 51′ 36.0″ E	8–20 m<br>reef slope	ND	red colored surface, slightly calcified and paper like texture.
**G1302**		Munda, New Georgia, Solomon Islands	8° 20′ 30″ S<br>157° 12′ 26″ E	7–28 m<br>reef slope	common	dark red/purple colored surface with greyish green underside, about 25%

						epibiont coverage including sponge, crustose, and calcified texture.
**G1587**	**2017**	Uepi, New Georgia, Solomon Islands	8° 26′ 33″ S 157° 56′ 42″ E	15–25 m under underhang	common	maroon colored surface with greyish underside, broad blade, lightly calcified, pliable.
**G1588**	**2017**	Uepi, New Georgia, Solomon Islands	8° 26′ 33″ S 157° 56′ 42″ E	15–25 m under underhang	common	maroon colored surface, broad blade, strongly calcified, breaks like potato chips.
**G1660**	**2017**	Nacula Island, Fiji	16° 51′ 03″ S 177° 24′ 11″ E	10–20 m	rare	maroon colored surface, 1–8 cm wide lobes, about 25% epibiont coverage including sponge, encrusting and slightly calcified texture.
**G1661**	**2017**	Nacula Island, Fiji	16° 51′ 03″ S 177° 24′ 11″ E	10–20 m reef slope	rare	pinkish maroon colored surface, 1–8 cm wide lobes, about 25% epibiont coverage including sponge, encrusting and very hard/crunchy texture.
**G1804**	**2018**	Namara passage, Kadavu Island, Fiji	18° 51′ 47.5″ S 178° 27′ 00.0″ E	10 m under overhang	ND	maroon colored surface with some yellow mustard streaks, about 5 cm wide blades, medium hard and crunchy texture.
**G1805**	**2018**	Namara passage, Kadavu Island, Fiji	18° 51′ 47.5″ S 178° 27′ 00.0″ E	subtidal	ND	reddish/maroon colored surface, about 5 cm wide blades, about 25% epibiont coverage, hard and crunchy texture.
**G1814**	**2018**	Dravuni Island, Fiji	18° 45′ 43″ S 178° 28′ 07″ E	15 m reef slope, underhang	common	maroon colored surface with orange rim and beige underside, about 2–10 cm wide blades, about 25% epibiont coverage, fouled on undeside, hard and leathery texture.
**G1815**	**2018**	Vanuakula island, Fiji	18° 42′ 29″ S 178° 29′ 54″ E	15 m reef slope, underhang	common	maroon colored surface with orange rim and beige underside, about 2–10 cm wide blades, about 25% epibiont coverage, fouled on undeside, hard and leathery texture.
**G1826**	**2018**	Nabouwalu, Ono Island, Fiji	18° 52′ 59″ S 178° 27′ 36″ E	10–15 m reef slope	common	maroon colored surface with orange rim and beige underside, about 2–10 cm wide blades, fouled on undeside, hard and leathery texture.
**G1834**	**2018**	Nabouwalu, Ono Island, Fiji	18° 52′ 47″ S 178° 26′ 40″ E	13–18 m reef slope, under ledges	rare	maroon colored surface with some yellow mustard streaks, about 25% epibiont coverage in underside, calcified and crunchy texture.
**G1835**	**2018**	Nabouwalu, Ono Island, Fiji	18° 52′ 47″ S 178° 26′ 40″ E	13–18 m reef slope, under ledges	common	maroon colored surface with orange rim, about 10 cm wide blades, hard and leathery texture.
**G1836**	**2018**	Nabouwalu, Ono Island, Fiji	18° 52′ 47″ S 178° 26′ 40″ E	13–18 m reef slope	ND	maroon colored surface, about 2–6 cm wide blades, some epibiont coverage on underside, hard and leathery texture.
**G1837**	**2018**	Nabouwalu, Ono Island, Fiji	18° 53′ 01″ S 178° 27′ 38″ E	12–14 m reef slope	abundant	maroon colored surface, about 2–10 cm wide blades,

						about 25% epibiont coverage on underside, medium hard and leathery texture.
**G1840**	**2018**	Nabouwalu, Ono Island, Fiji	18° 52′ 14″ S<br>178° 29′ 38″ E	8 m	ND	maroon colored surface, about 2–10 cm wide blades, about 25% epibiont coverage on underside, medium hard and leathery (comparatively crispy) texture.

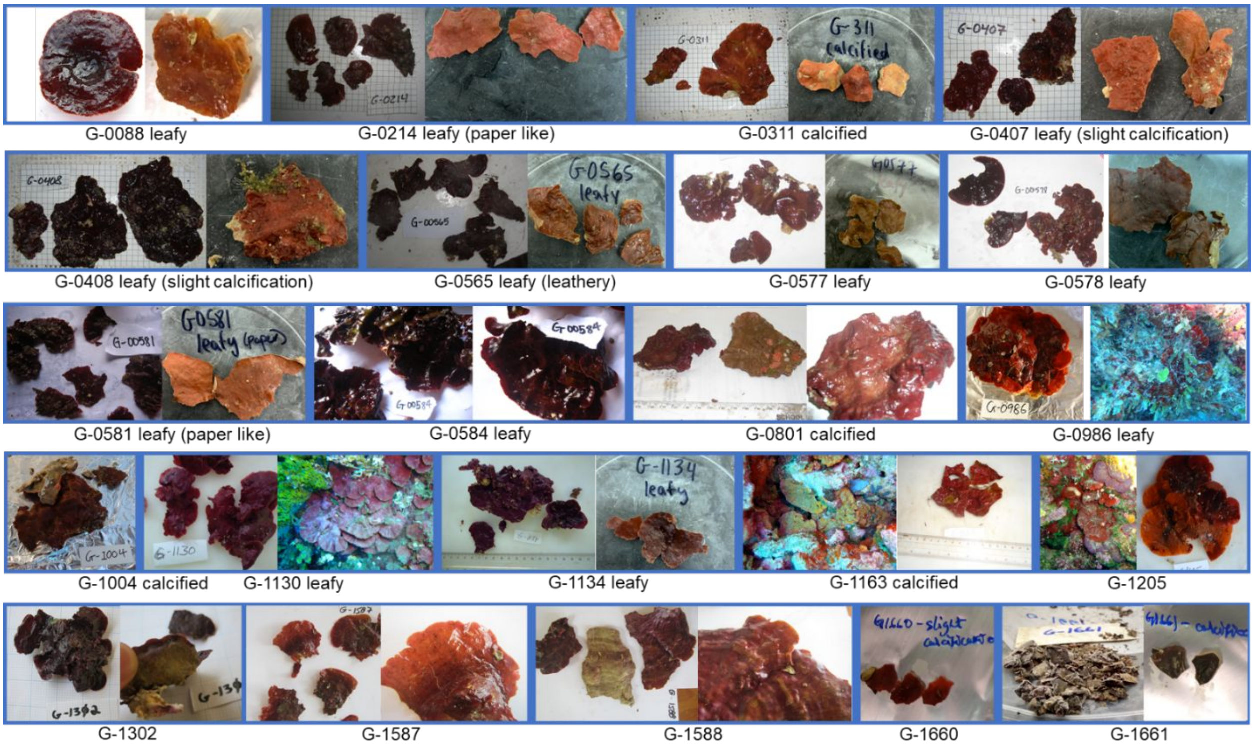

Figure S1. Images for Fijian and the Solomon Islands collections (generated during field collection or taken for algal samples in the lab after thawing collections preserved at –80 °C) of *Peyssonnelia* spp. depicting leafy, leathery, and crunchy (calcified) consistency. Photographs courtesy of M.E. Hay. Copyright 2023.

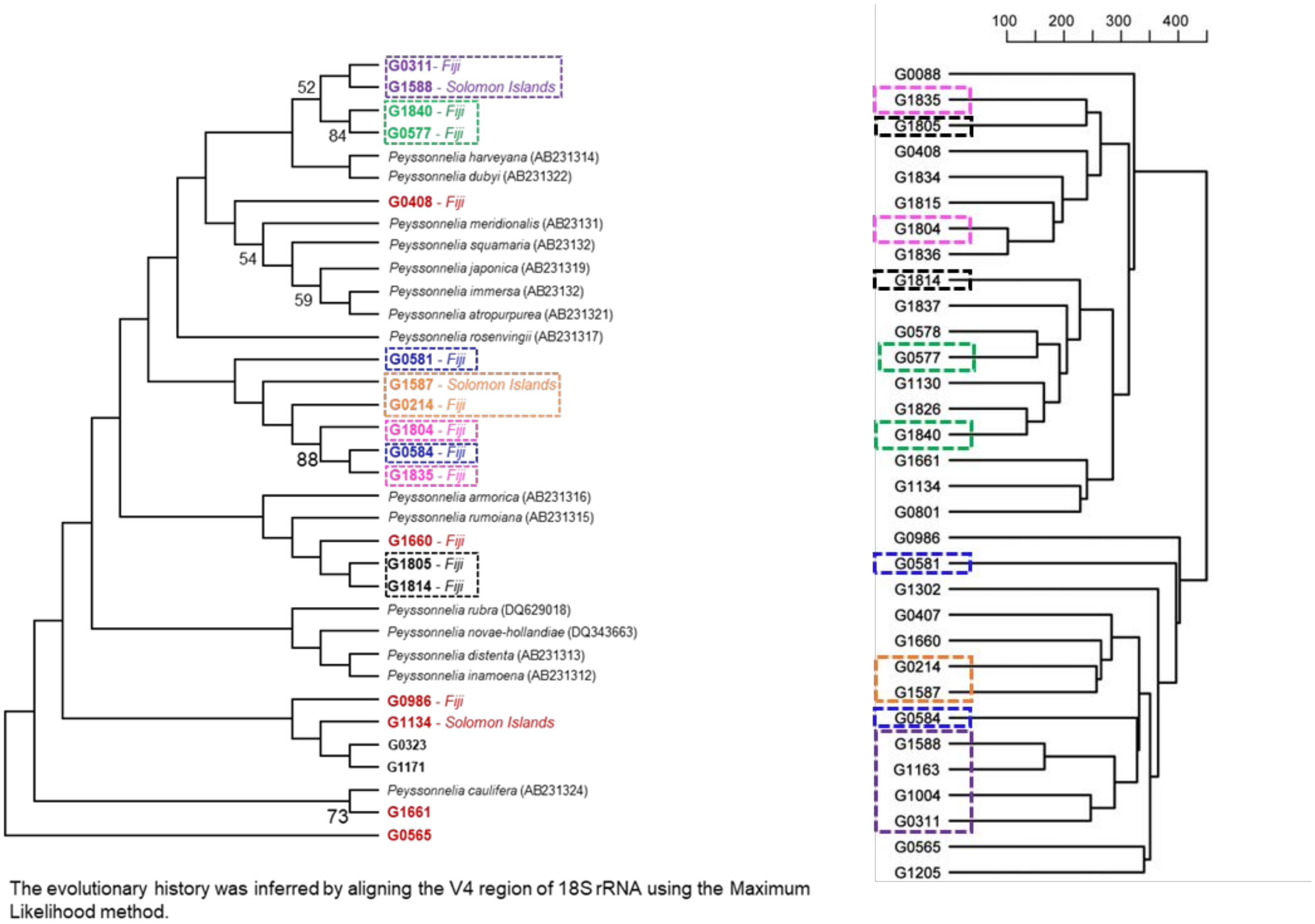

Figure S2. **Left**: Evolutionary relationship of 18 *Peyssonnelia* spp. samples (of 32 studied) with related red algal species from family Peyssonneliaceae inferred by aligning the V4 region of 18S small subunit (SSU) rRNA sequences using the Maximum Likelihood method. **Right:** Correlations observed between phylogeny and hierarchical cluster analysis based on negative ionization mode LC-MS data.

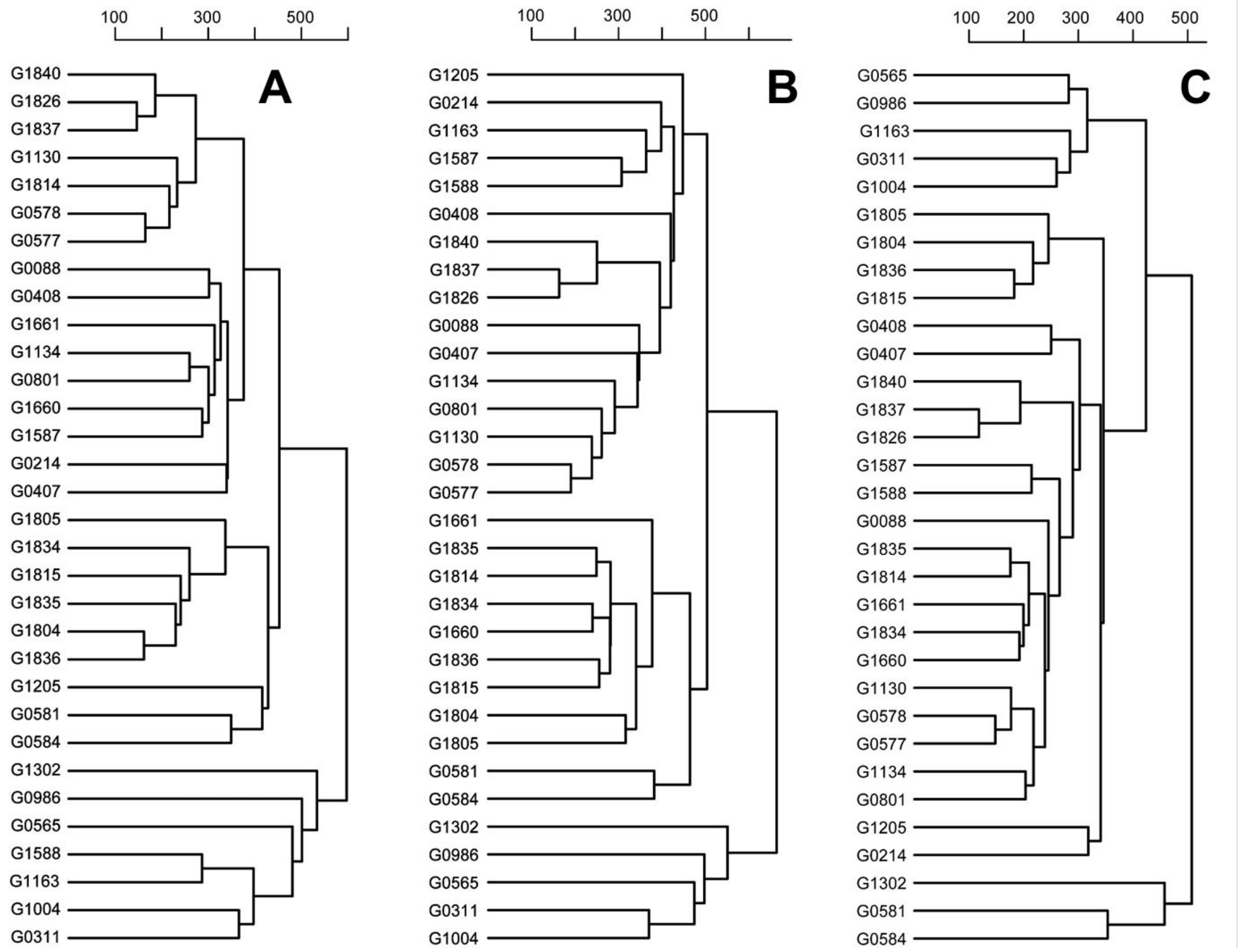

Figure S3. Hierarchical cluster analysis for **A**: Positive ionization mode HRMS data acquired for mid-polarity fraction B by HP20SS fractionation. **B**: Positive ionization mode HRMS data, and **C**: Negative ionization mode HRMS data acquired for mid-polarity fraction C by HP20SS fractionation.

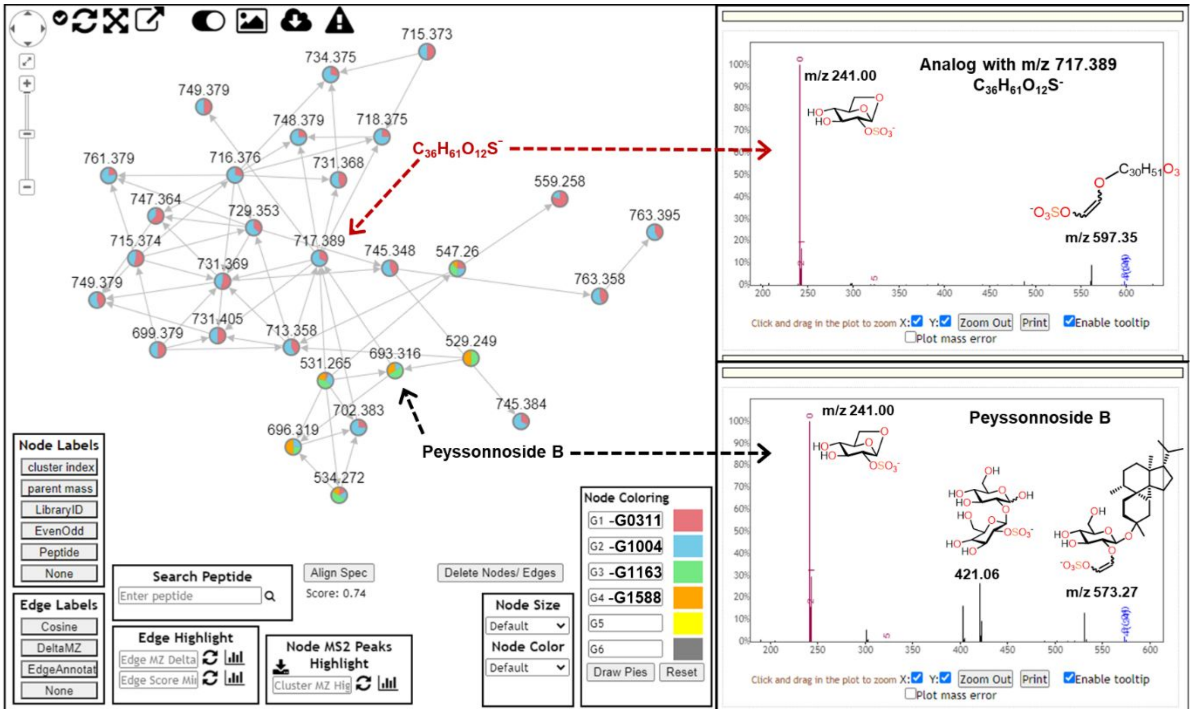

Figure S4. Comparison of MS[2] fragmentation pattern of peyssobaricanoside **B**–**C** (**16**–**17**, *m/z* 717.389) with that of peyssonnoside B (**2**). The fragment ion with *m/z* 241 indicates loss of the sulfated monosaccharide. Fragment ion with *m/z* 597 as observed for the molecular ion peak *m/z* 717.389 and fragment ion with *m/z* 573 as observed for **2** arose from a retro-Diels-Alder based fragmentation whereby a neutral fragment of 118 amu is lost.

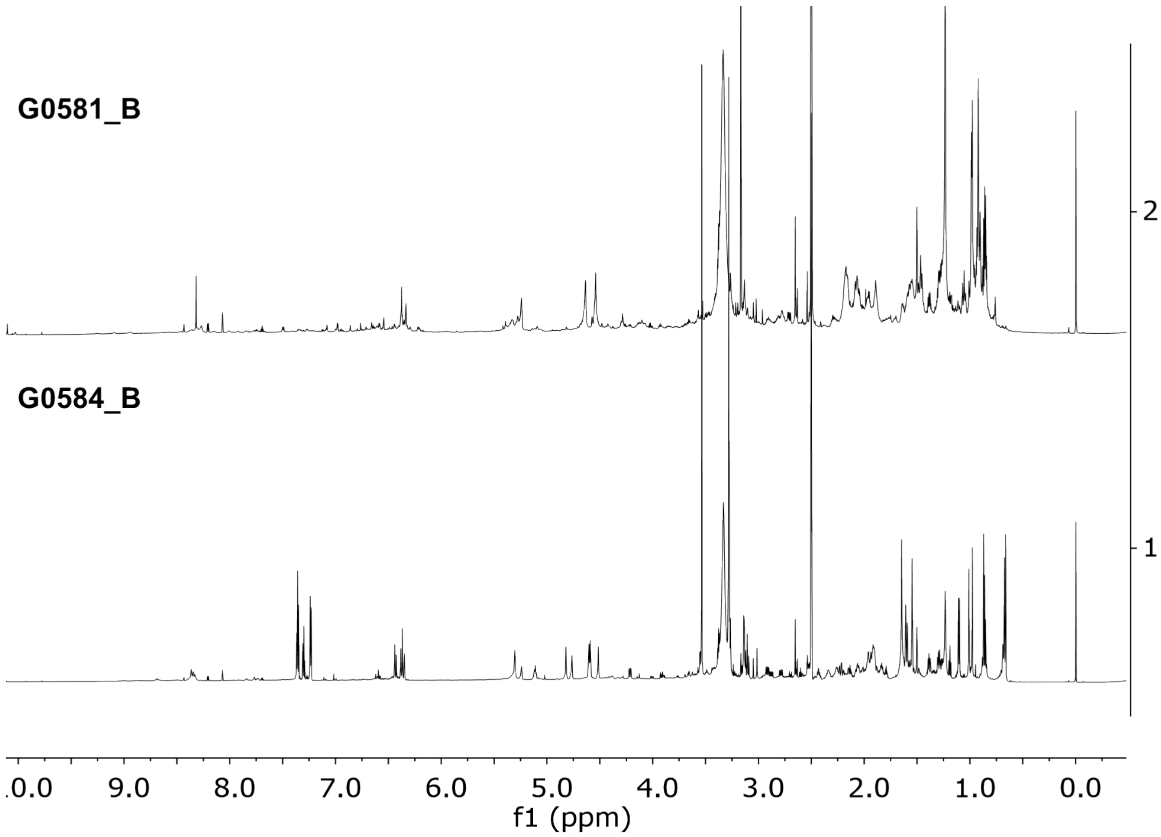

Figure S5. Overlay of ^{1}H NMR spectroscopic data for mid-polarity HP20SS fraction B of G0584 and G0581 in DMSO-d_6 (800MHz).

Table S2. Solvent gradient used for HPLC separation of G0311

Time	A	B	C	D
0	5	0	45	50
3	5	0	45	50
22.8	5	85.8	4.4	4.8
25	5	0	45	50
30	5	0	45	50

A: water (2% HCOOH), B: isopropanol, C: water, D: acetonitrile, flow rate: 0.8 ml/min

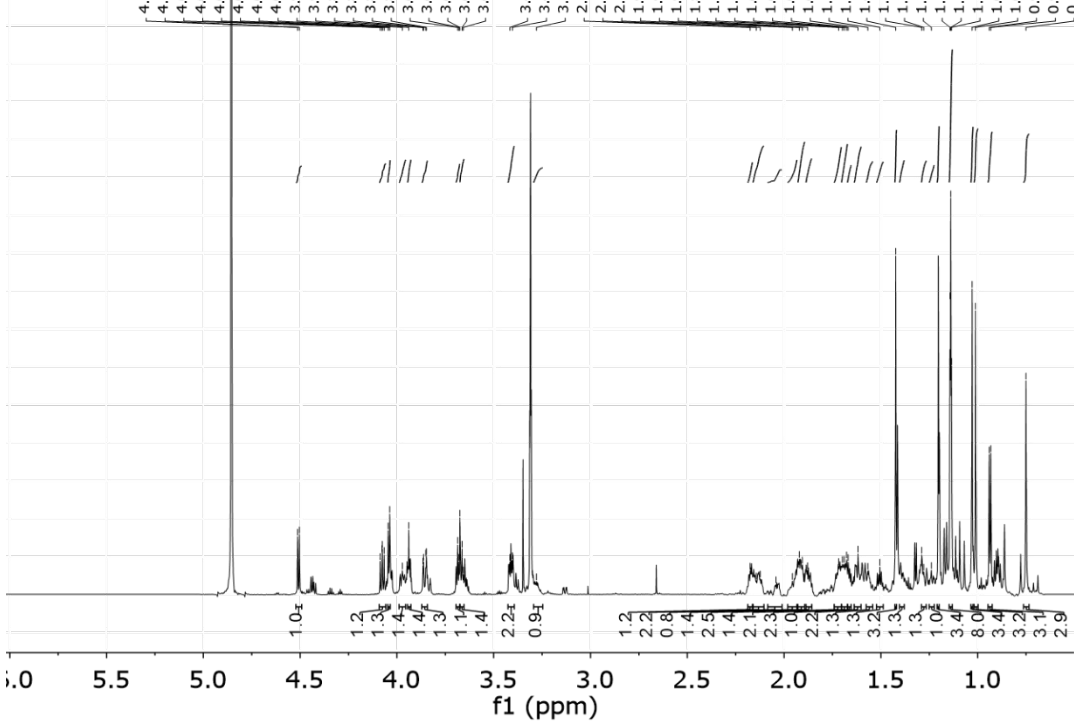

Figure S6. ^{1}H NMR spectrum for peyssobaricanoside A (**15**) in CD_3OD (800 MHz)

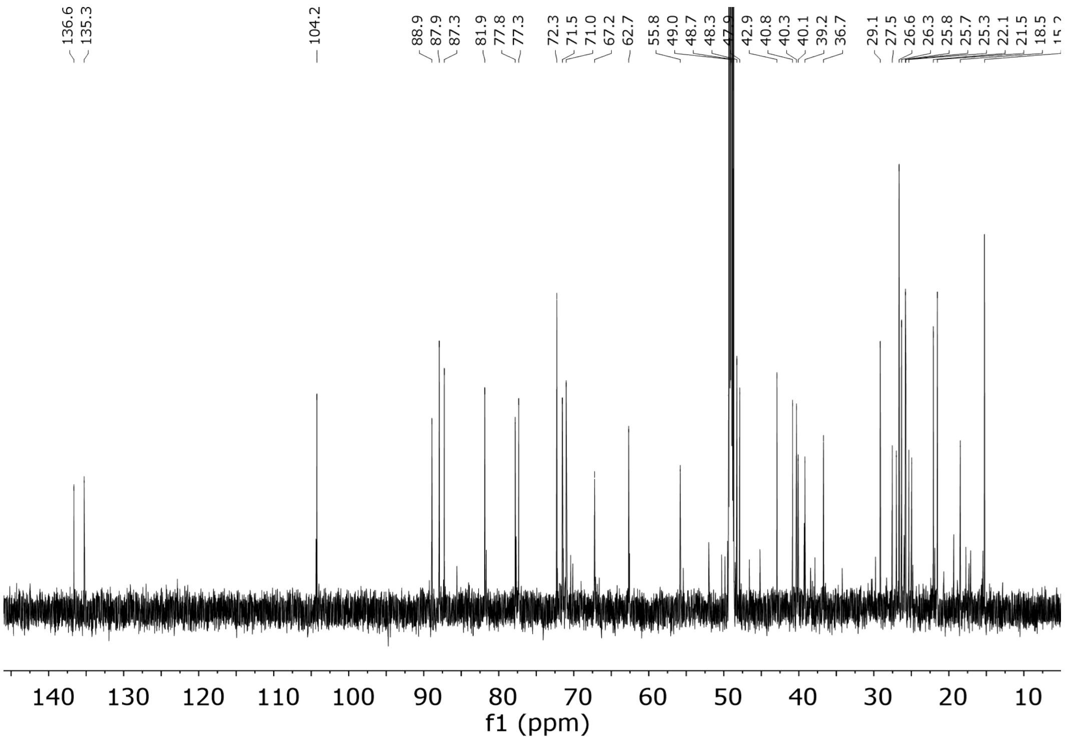

Figure S7. ^{13}C NMR spectrum for peyssobaricanoside A (**15**) in CD_3OD (800 MHz)

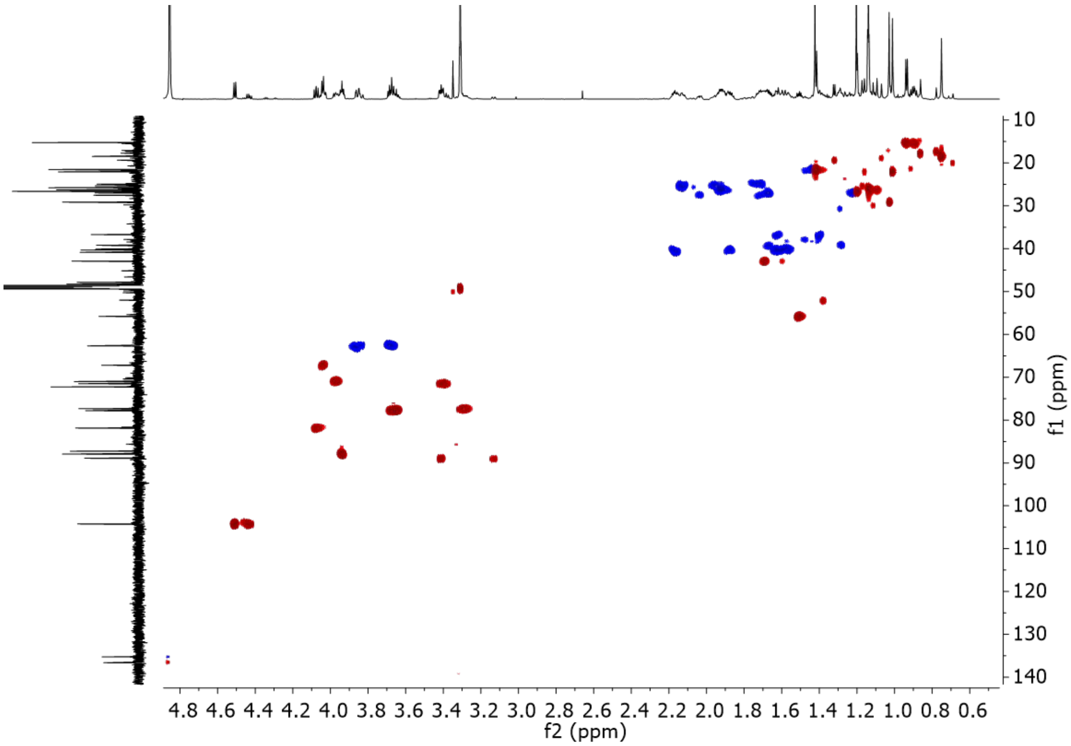

Figure S8. HSQC NMR spectrum for peyssobaricanoside A (**15**) in CD_3OD (800 MHz)

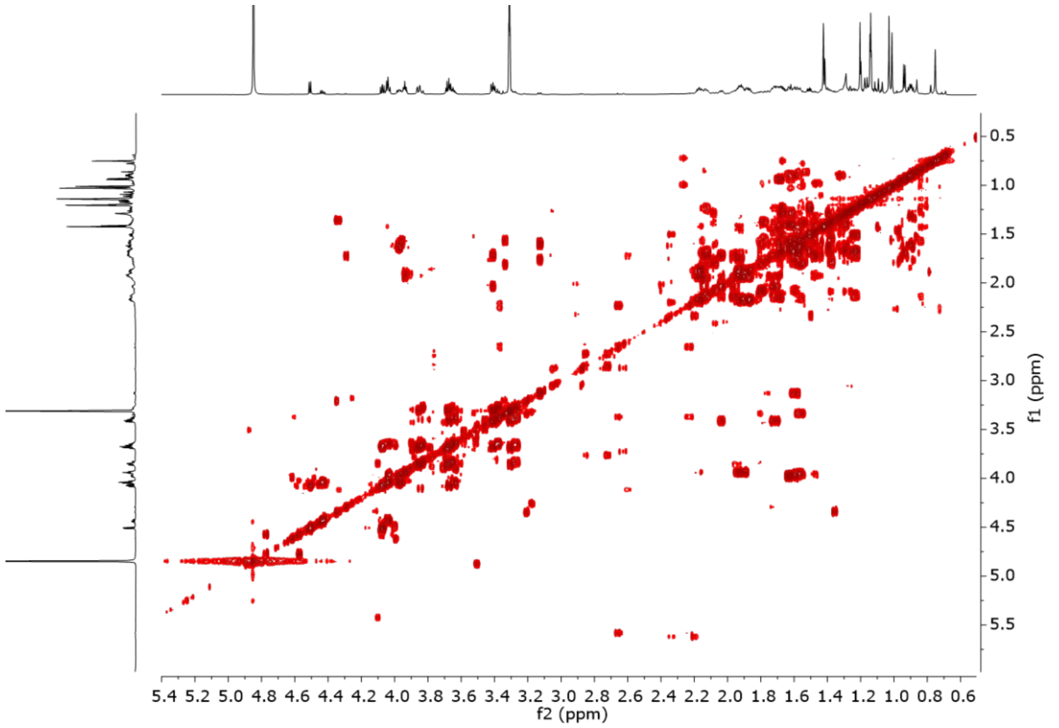

Figure S9. COSY NMR spectrum for peyssobaricanoside A (**15**) in CD_3OD (800 MHz)

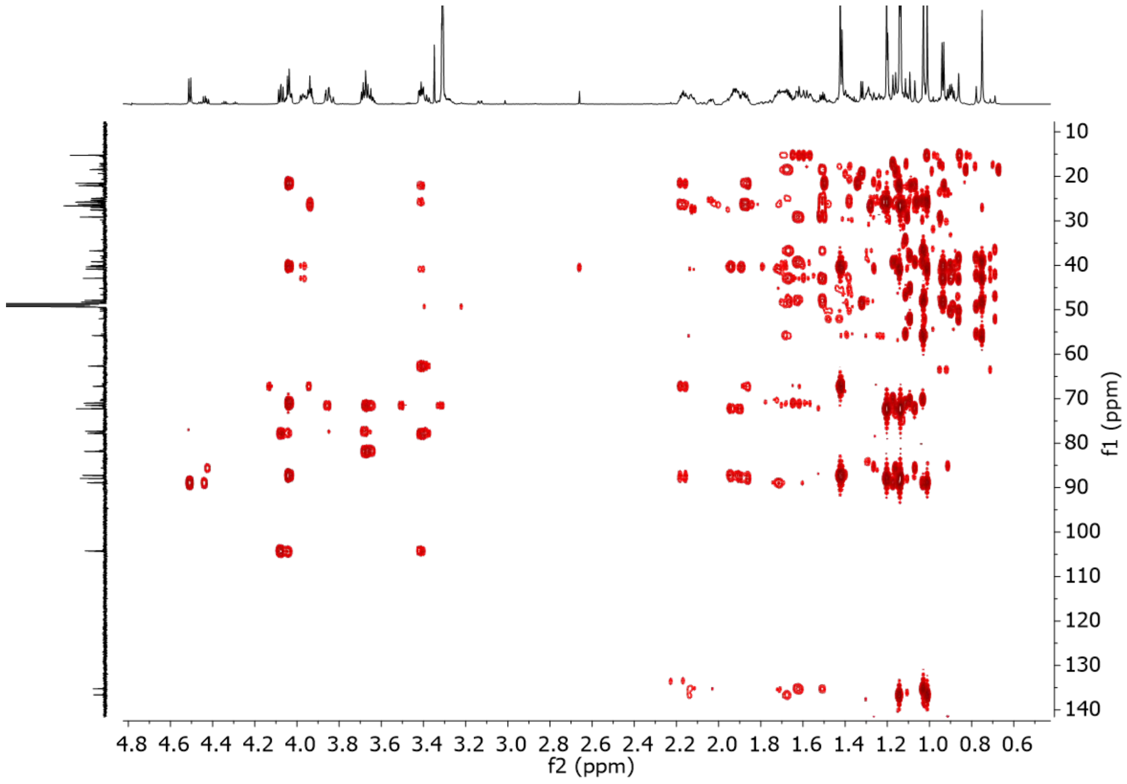

Figure S10. HMBC NMR spectrum for peyssobaricanoside A (**15**) in CD_3OD (800 MHz)

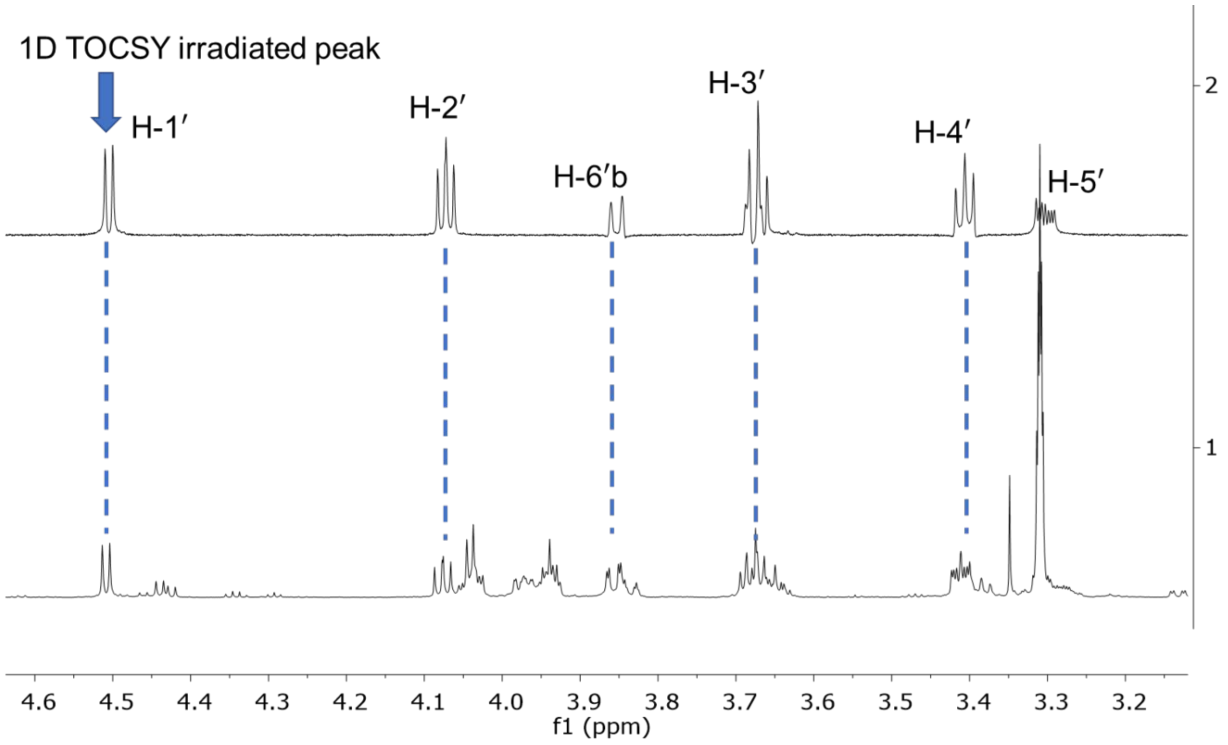

Figure S11. 1D TOCSY (H-1′ irradiated) NMR spectrum for peyssobaricanoside A (**15**) in CD_3OD (800 MHz)

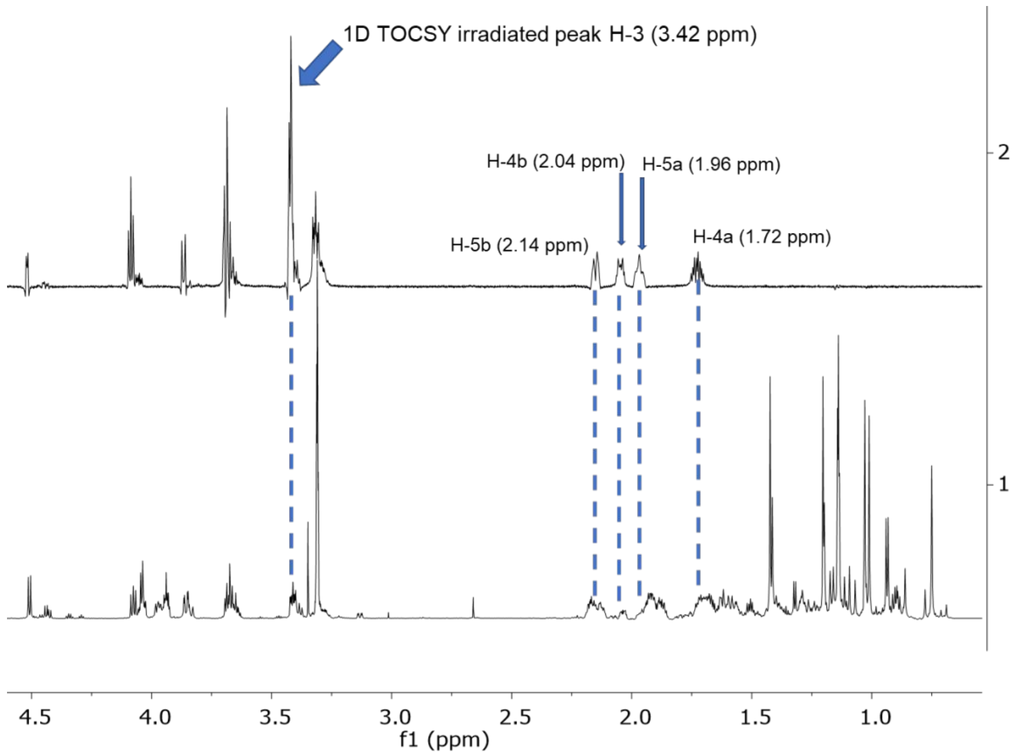

Figure S12. 1D TOCSY (H-3 irradiated) NMR spectrum for peyssobaricanoside A (**15**) in CD_3OD (800 MHz)

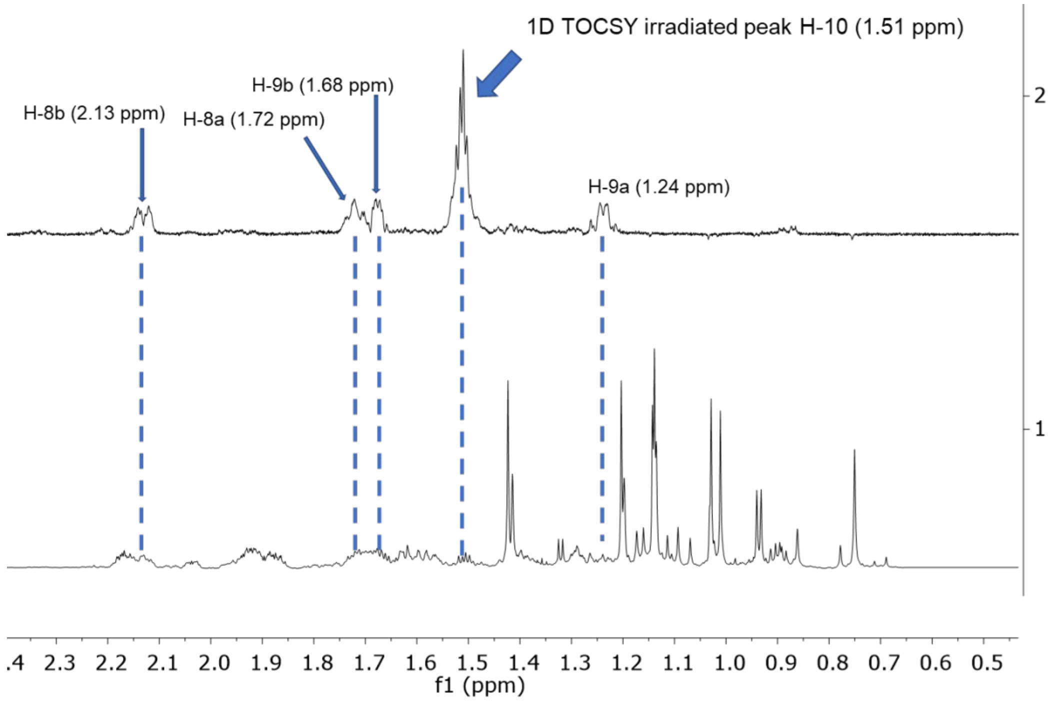

Figure S13. 1D TOCSY (H-10 irradiated) NMR spectrum for peyssobaricanoside A (**15**) in CD_3OD (800 MHz)

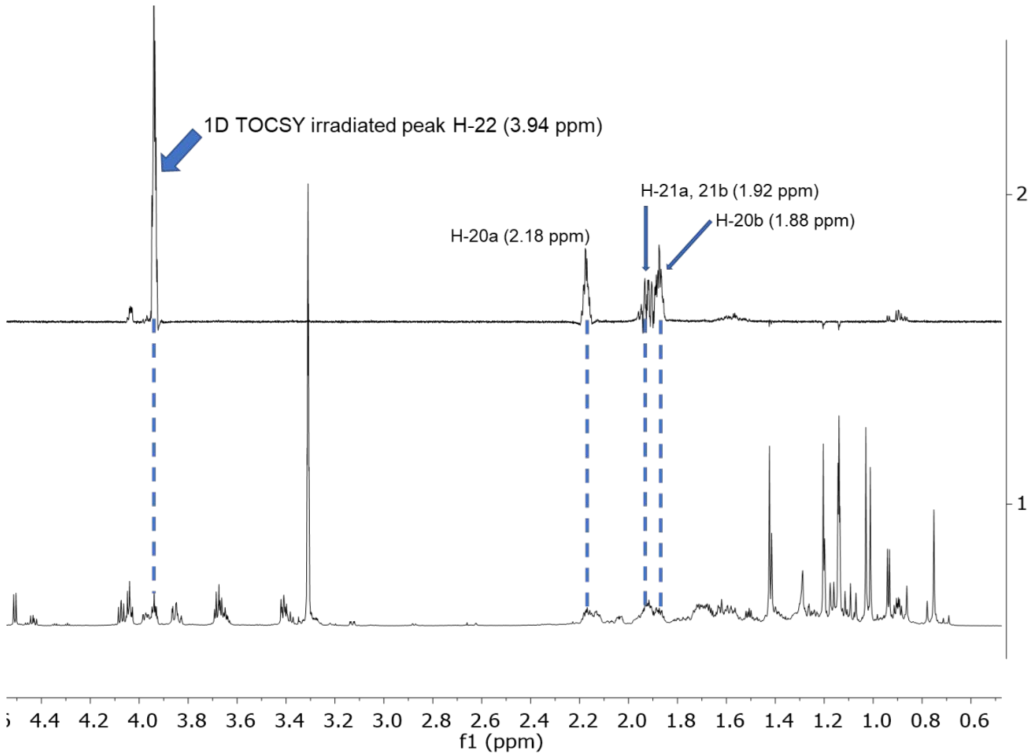

Figure S14. 1D TOCSY (H-22 irradiated) NMR spectrum for peyssobaricanoside A (**15**) in CD_3OD (800 MHz)

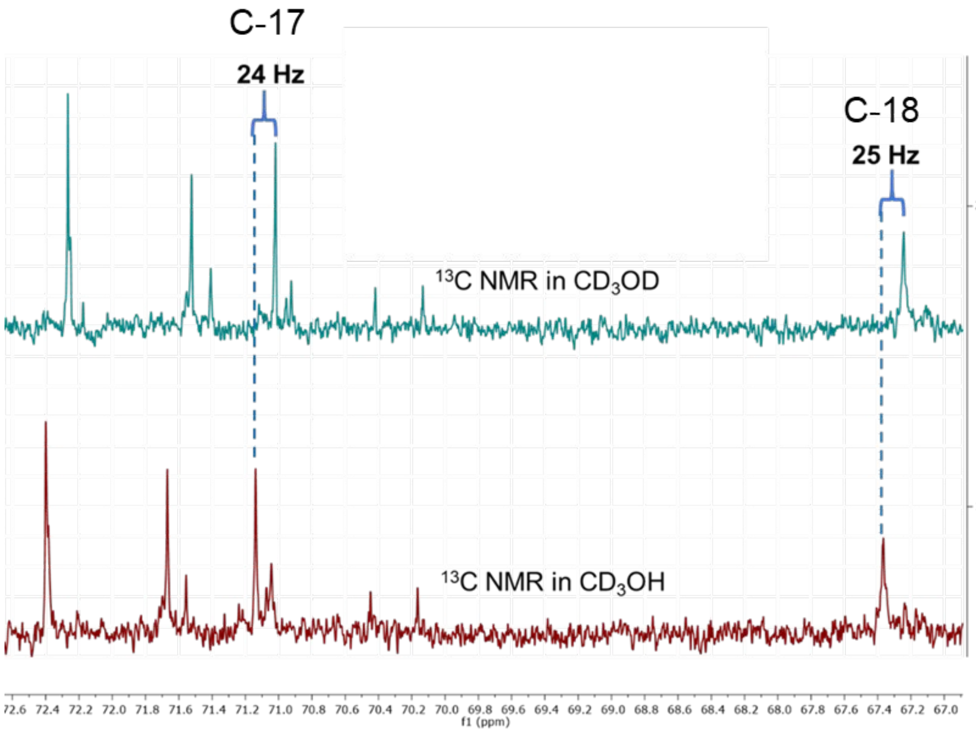

Figure S15. Difference in ^{13}C NMR chemical shift for peyssobaricanoside A (**15**) when acquired in CD_3OD vs CD_3OH (solvent isotope effect in ^{13}C NMR chemical shift).

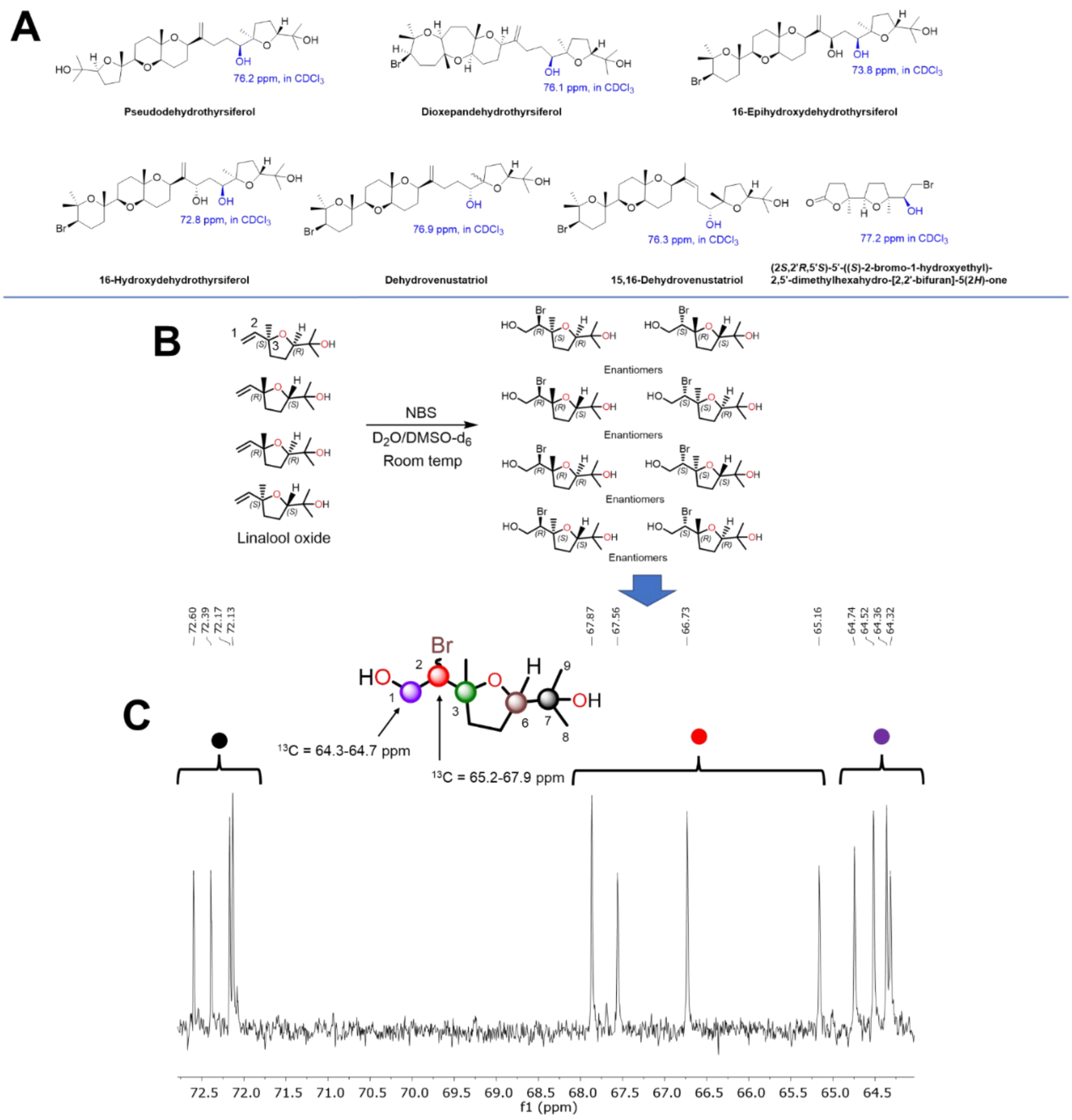

Figure S16. (**A**) ^{13}C NMR chemical shifts for a hydroxyl-containing carbon adjacent to tetrahydrofuran ring (natural and synthetic compounds from existing literature).[1-3] (**B**) Reaction scheme showing the derivatization of linalool oxide (mixture) with *N*-bromosuccinimide (NBS) to generate halohydrin products (Note: The bromohydrin of linalool oxide was generated by reacting 4.8 mg of linalool oxide with 8.7 mg of NBS in 500 µl of D_2O:DMSO-d_6 (1:4) for 4 hours. Based on ^{1}H, ^{13}C, and HSQC NMR spectral data, the reaction was regioselective, likely due to steric effects generated by the methyl at C-3). (**C**) Interpretation of ^{13}C and HSQC NMR spectroscopic data showed that the bromine containing carbon (C-2), adjacent to the tetrahydrofuran ring had ^{13}C NMR shift 65.2–67.9 ppm. The opposite regiochemistry (bromide added to the C-1 group instead) would necessitate ^{13}C NMR chemical shifts around 30-40 ppm for C-1 which was not observed.

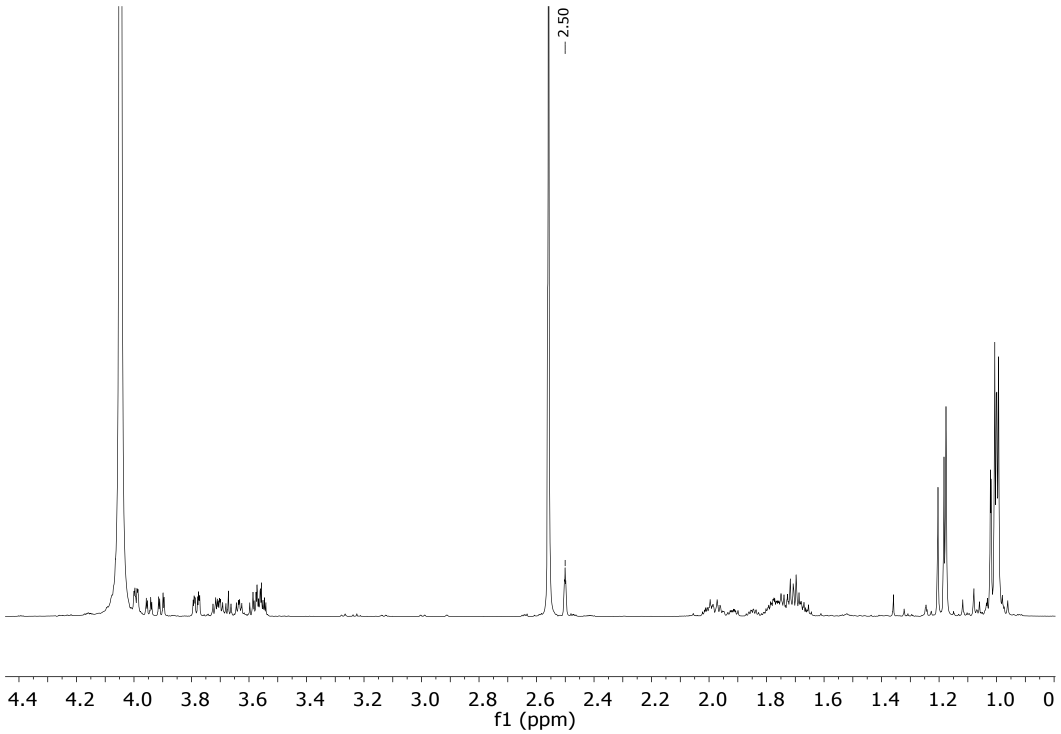

Figure S17. ^{1}H NMR spectrum for reaction of linalool-oxide with NBS as shown in **Figure S16**, **B** (D_2O:DMSO-d_6, 800 MHz)

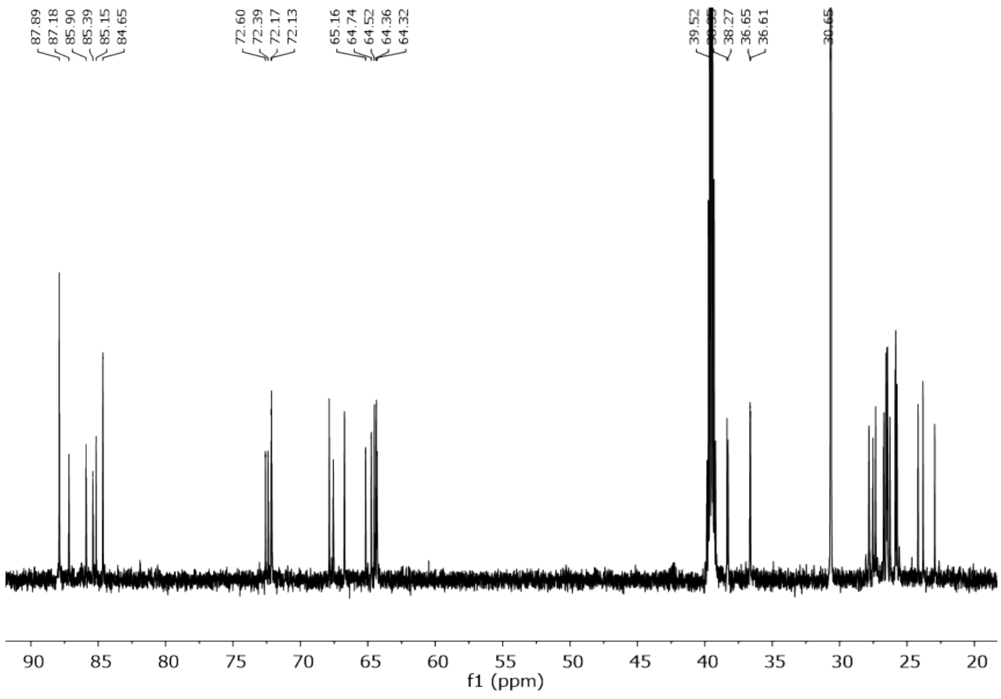

Figure S18. ^{13}C NMR spectrum for reaction of linalool-oxide with NBS as shown in **Figure S16**, **B** ((D_2O:DMSO-d_6, 800 MHz)

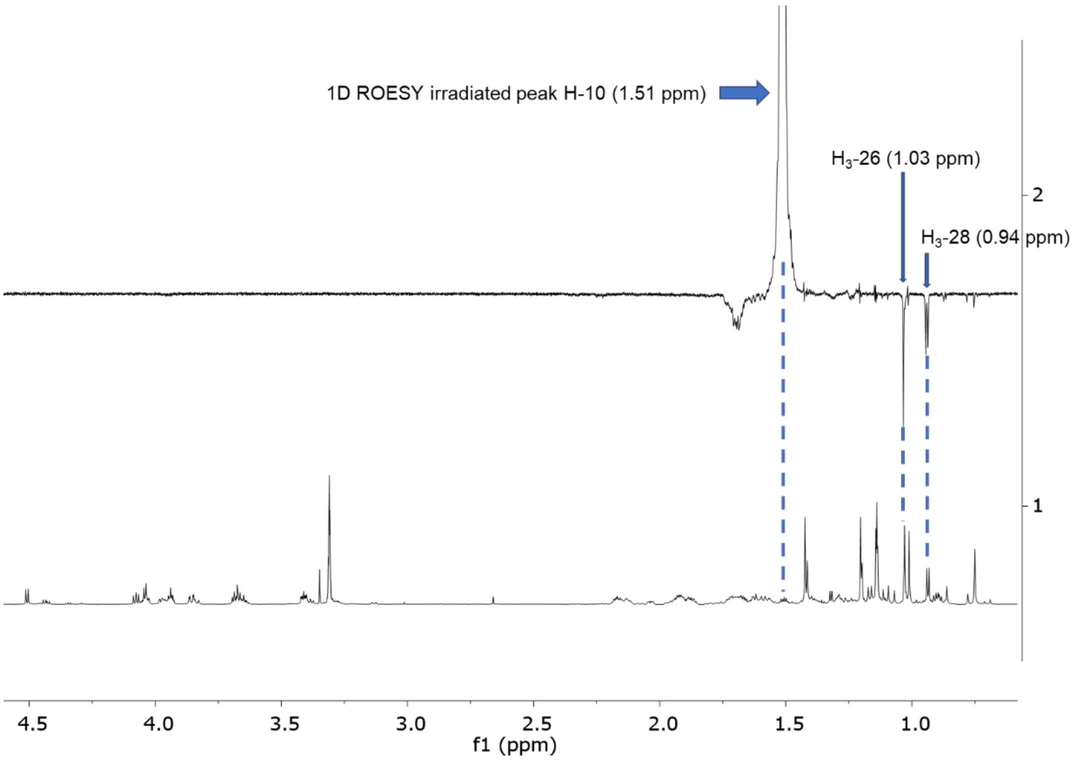

Figure S19. 1D ROESY (H-10 irradiated) NMR spectrum for peyssobaricanoside A (**15**) in CD_3OD (800 MHz)

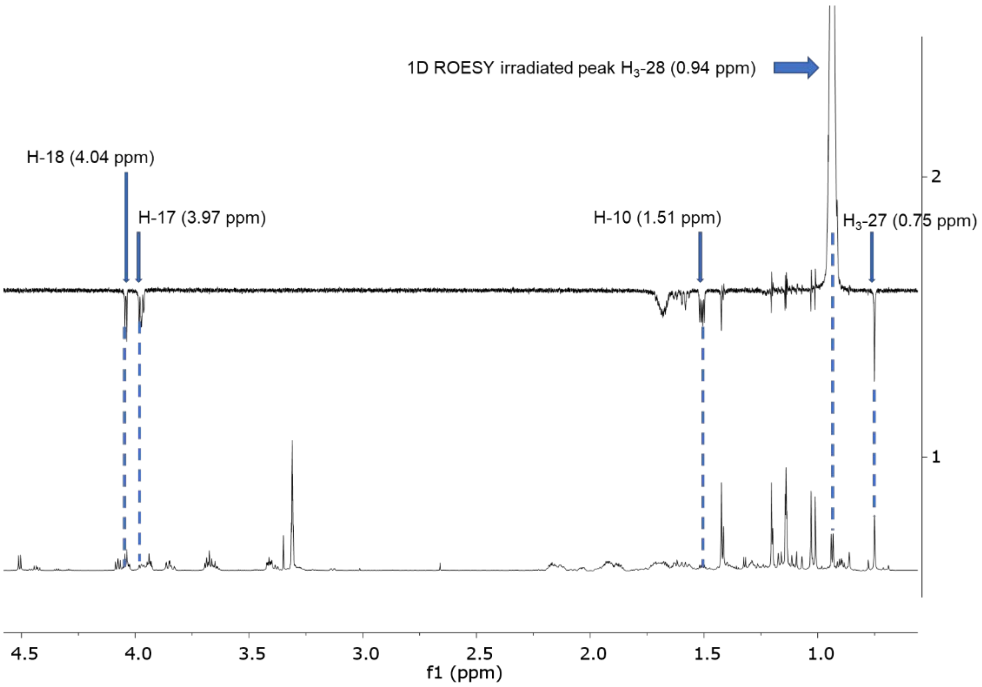

Figure S20. 1D ROESY (H_3-28 irradiated) NMR spectrum for peyssobaricanoside A (**15**) in CD_3OD (800 MHz)

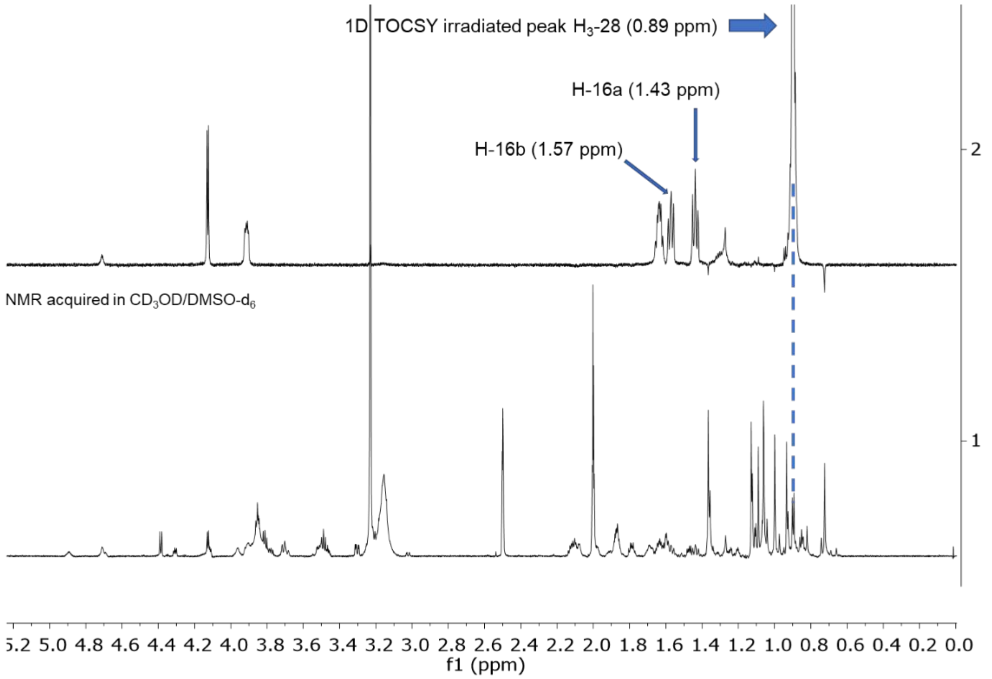

Figure S21. 1D TOCSY (H_3-28 irradiated) NMR spectrum for peyssobaricanoside A (**15**) in CD_3OD:DMSO-d_6 (800 MHz)

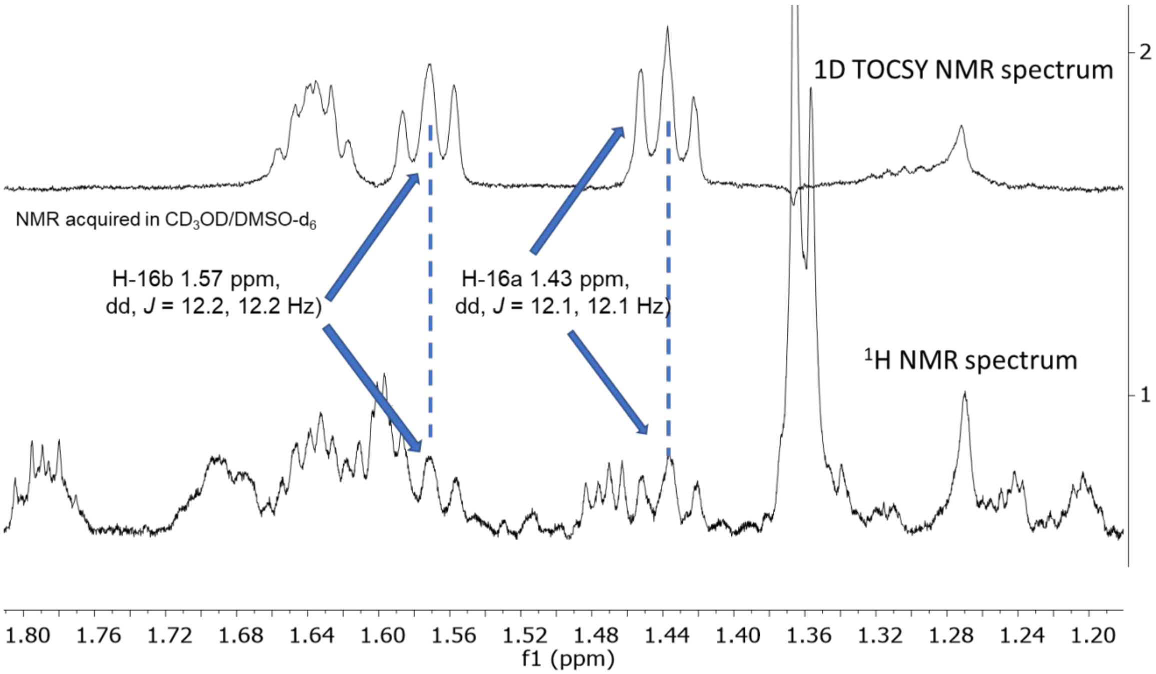

Figure S22. 1D TOCSY (H_3-28 irradiated) NMR spectrum for peyssobaricanoside A (**15**) in CD_3OD:DMSO-d_6 (800 MHz). Splitting pattern and *J* values for H-16a and H-16b were extracted from the ^{1}H NMR spectrum (bottom) and supported by the splitting observed in the 1D TOCSY spectrum (up).

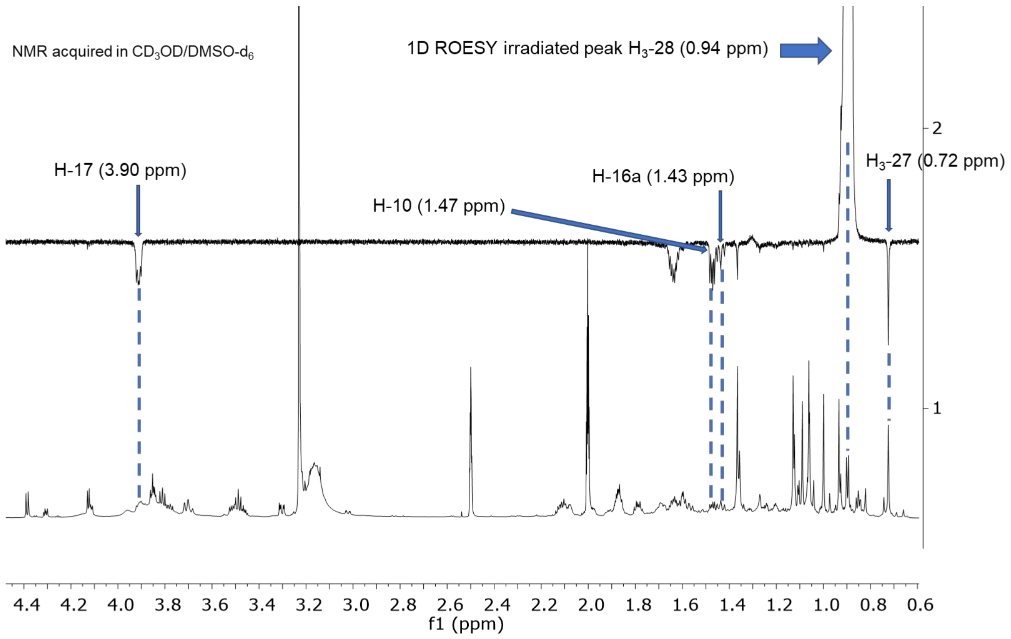

Figure S23. 1D ROESY (H_3-28 irradiated) NMR spectrum for peyssobaricanoside A (**15**) in CD_3OD:DMSO-d_6 (800 MHz)

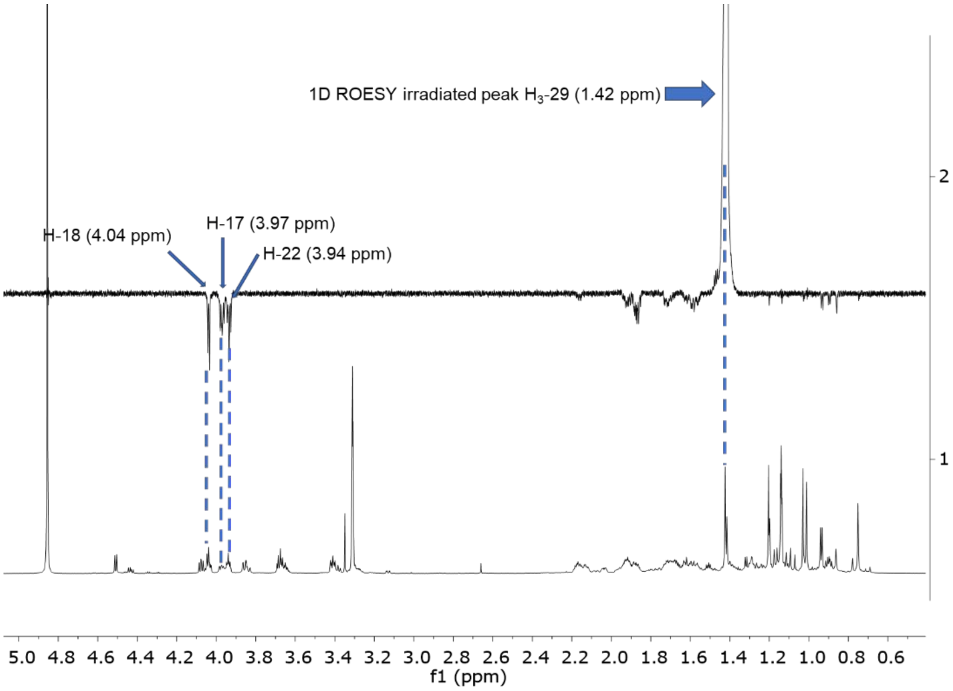

Figure S24. 1D ROESY (H_3-29 irradiated) NMR spectrum for peyssobaricanoside A (**15**) in CD_3OD (800 MHz)

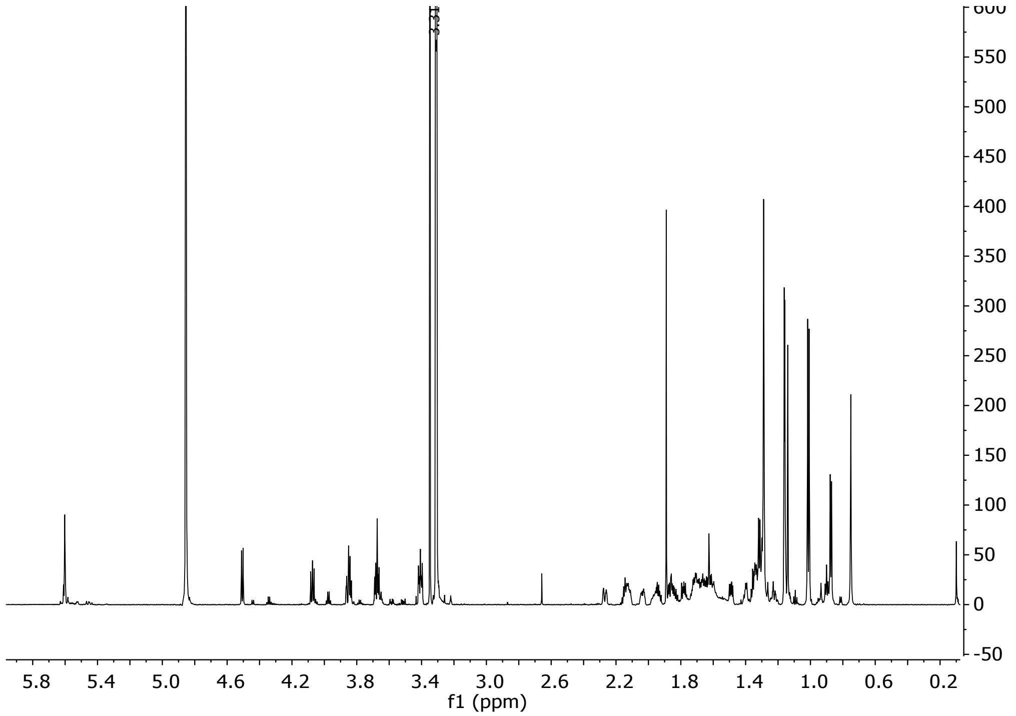

Figure S25. ^{1}H NMR spectrum for peyssobaricanoside B (**16**) in CD_3OD (800 MHz)

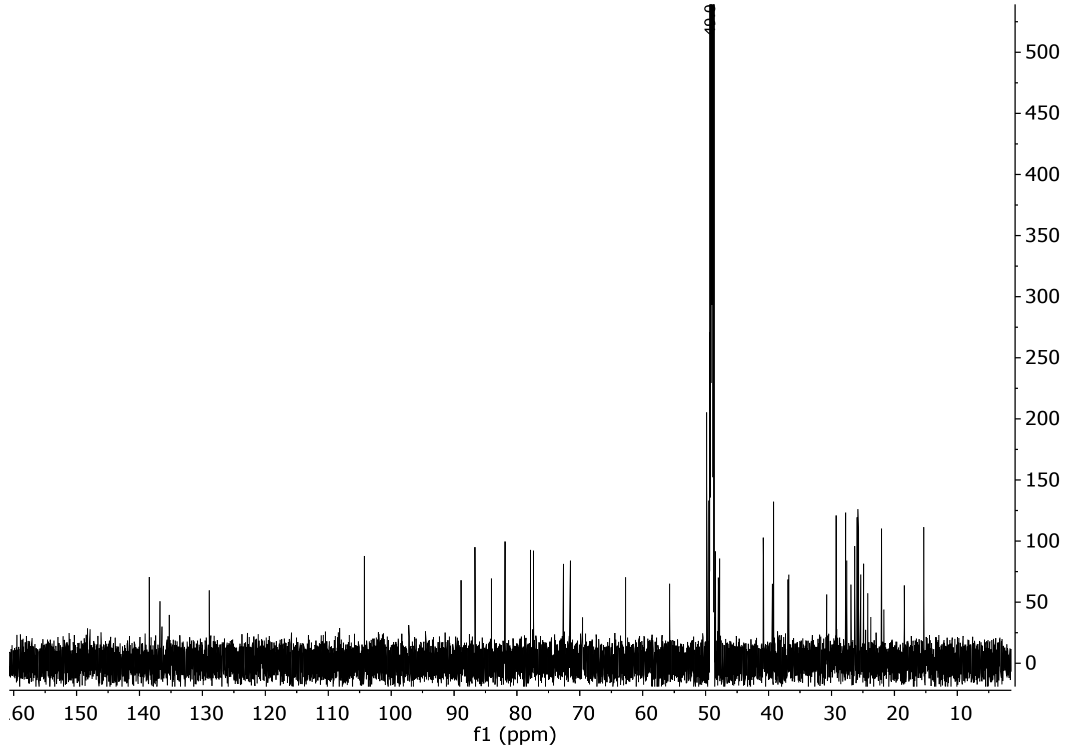

Figure S26. ^{13}C NMR spectrum for peyssobaricanoside B (**16**) in CD_3OD (800 MHz)

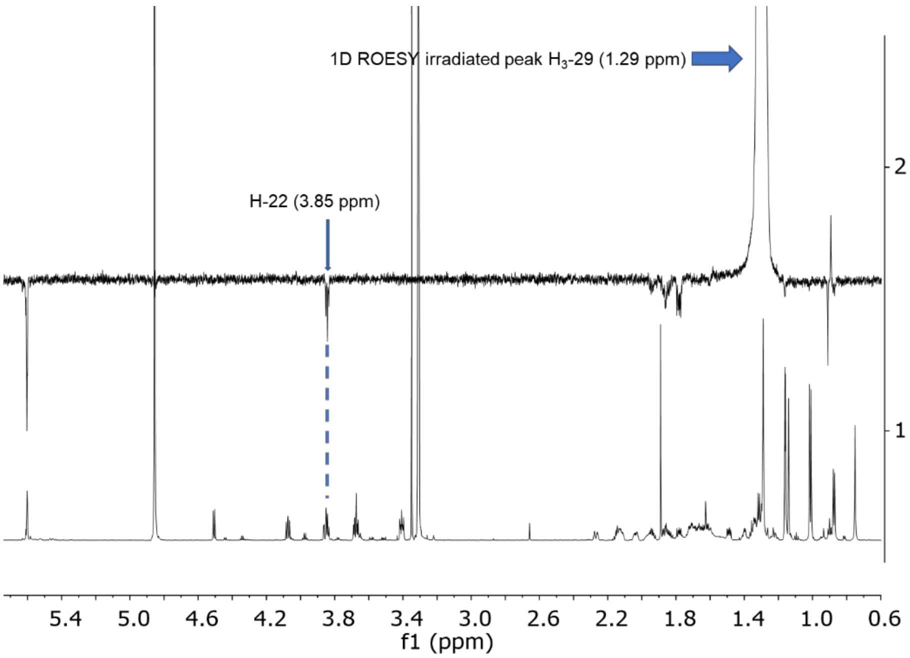

Figure S27. 1D ROESY NMR spectrum for peyssobaricanoside B (**16**) in CD_3OD (800 MHz).

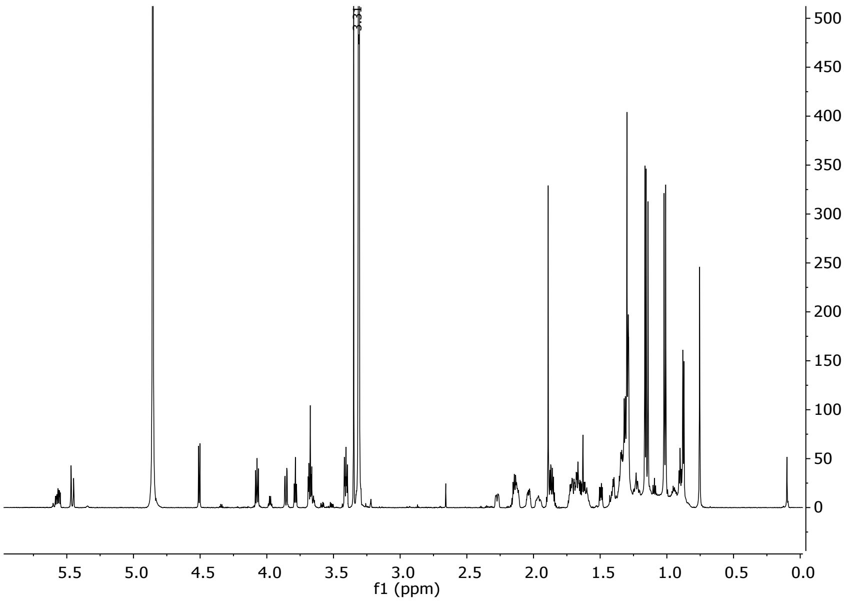

Figure S28. ^{1}H NMR spectrum for peyssobaricanoside C (**17**) in CD_3OD (800 MHz)

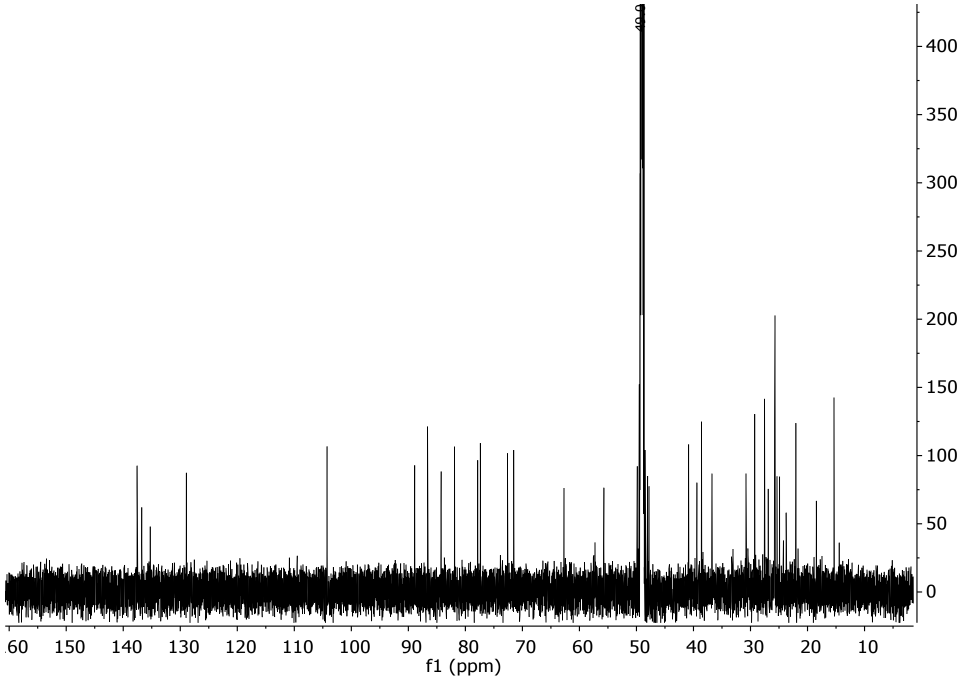

Figure S29. ^{13}C NMR spectrum for peyssobaricanoside C (**17**) in CD_3OD (800 MHz)

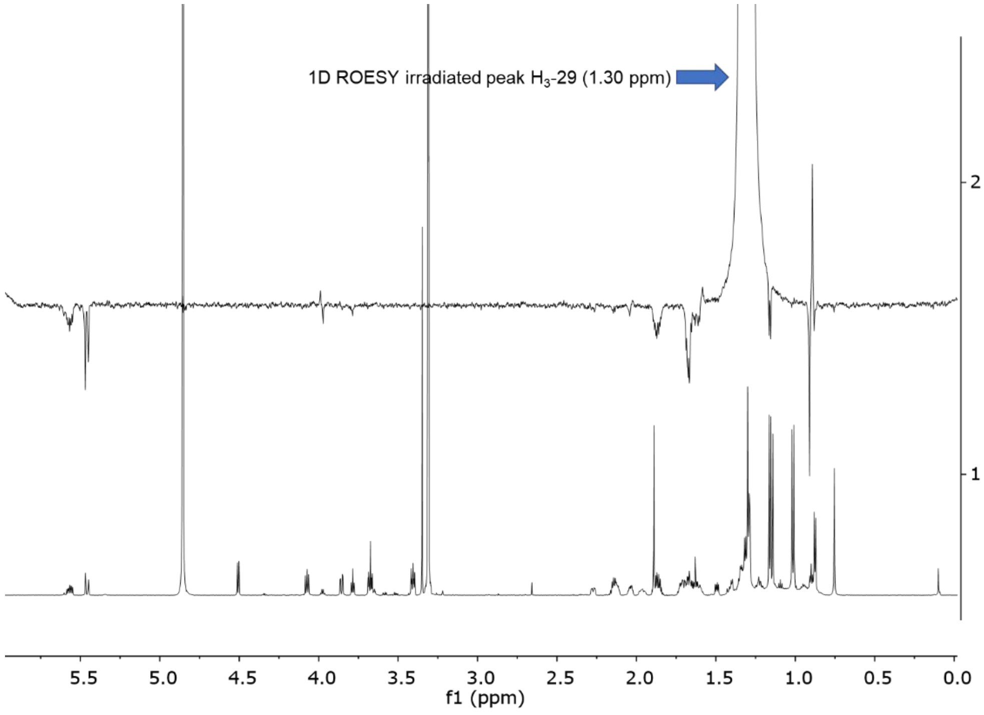

Figure S30. 1D ROESY NMR spectrum for peyssobaricanoside C (**17**) in CD_3OD (800 MHz). H_3-29 did not show a ROESY correlation with H-22.

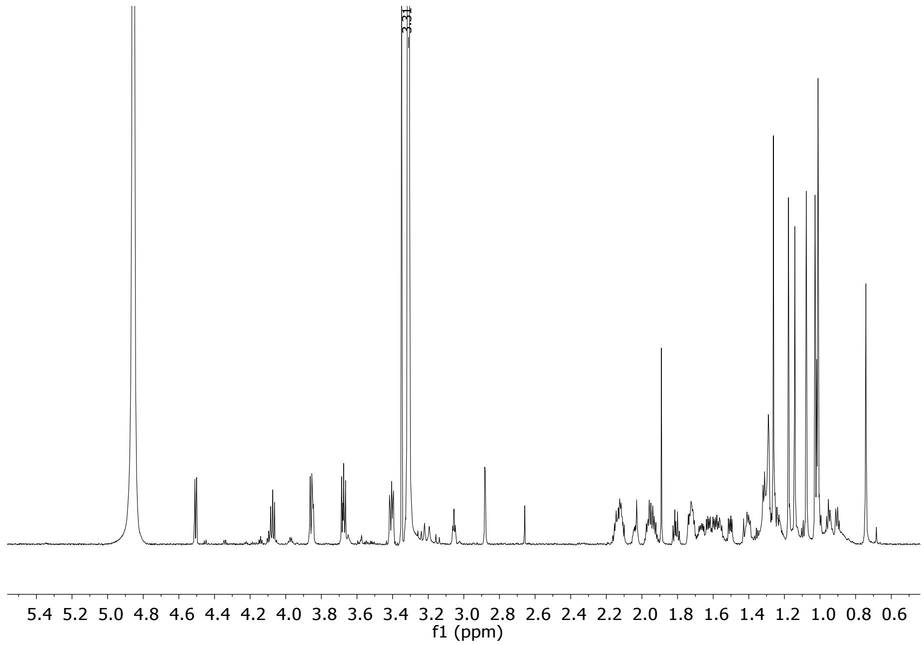

Figure S31. ^{1}H NMR spectrum for peyssobaricanoside D (**18**) in CD_3OD (800 MHz)

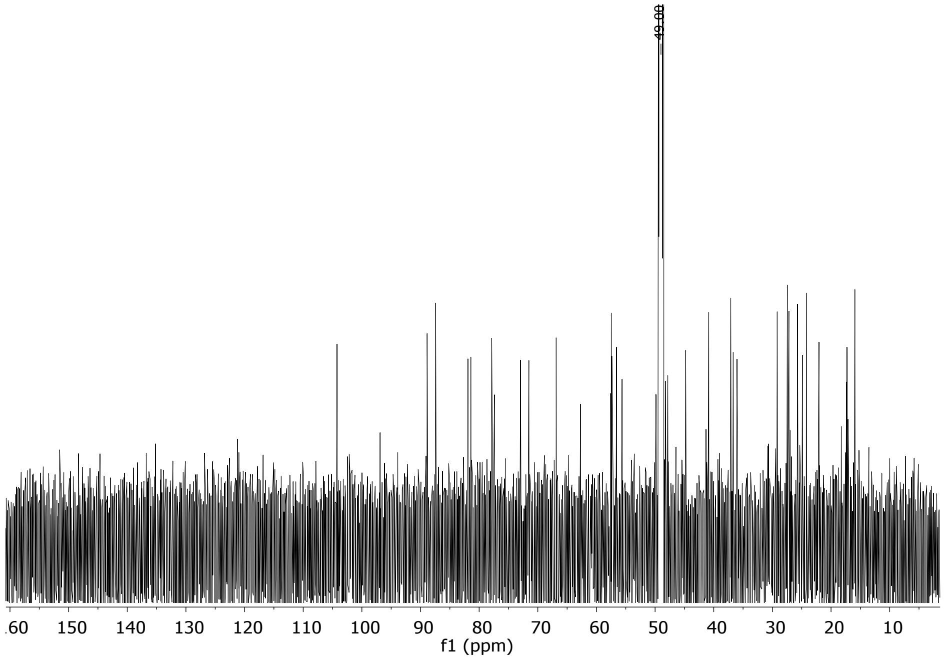

Figure S32. ^{13}C NMR spectrum for peyssobaricanoside D (**18**) in CD_3OD (800 MHz)

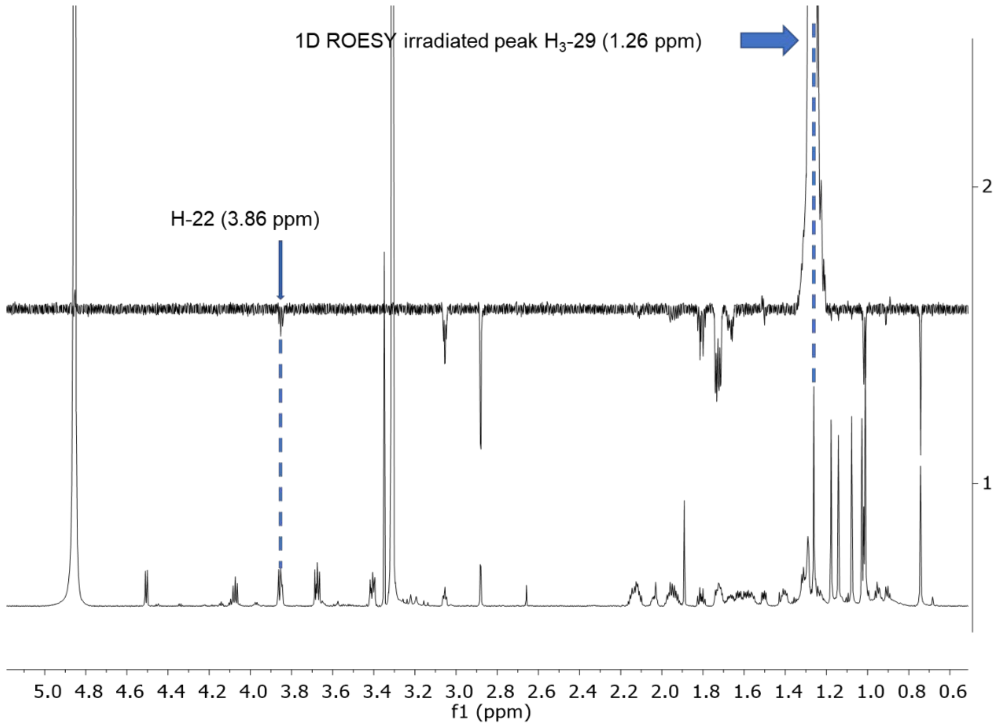

Figure S33. 1D ROESY NMR spectrum for peyssobaricanoside D (**18**) in CD_3OD (800 MHz).

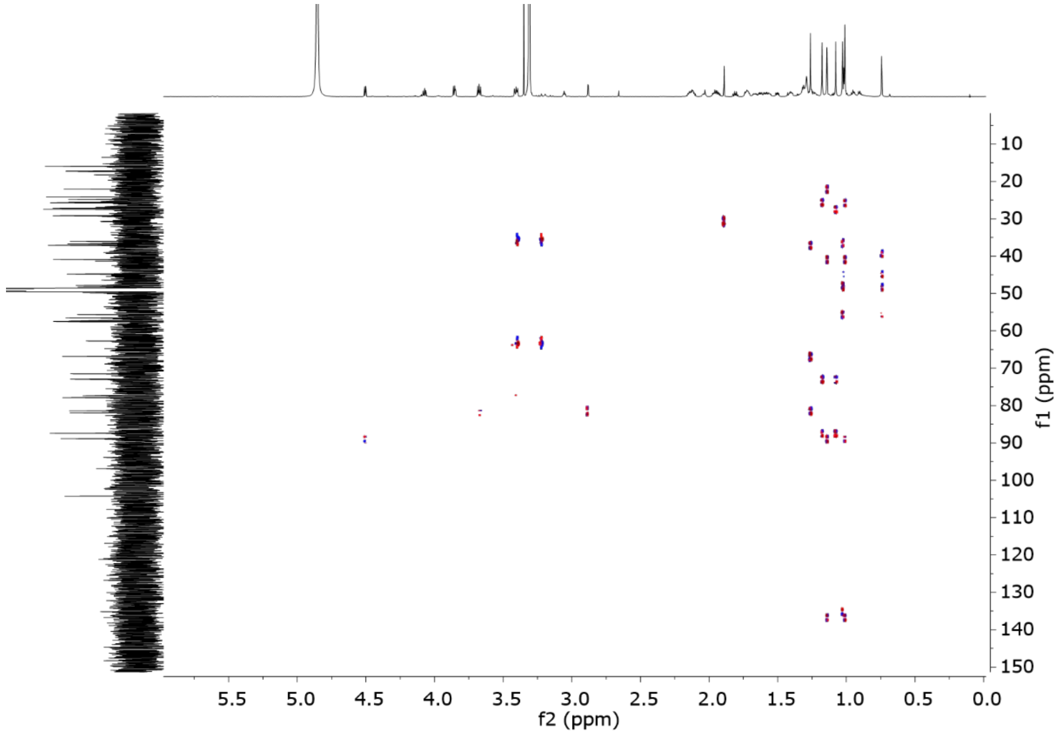

Figure S34. hmbcetgpjcl2nd NMR spectrum for peyssobaricanoside D (**18**) in CD_3OD (800 MHz).

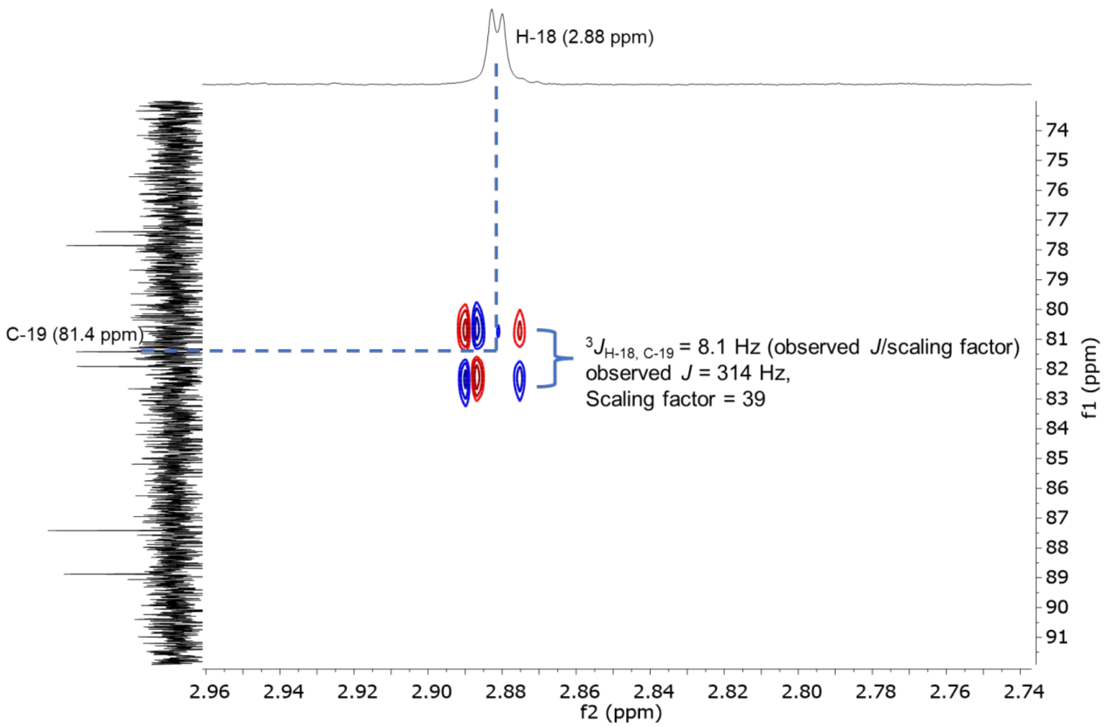

Figure S35. hmbcetgpjcl2nd NMR spectrum for peyssobaricanoside D (**18**) in CD_3OD (800 MHz).

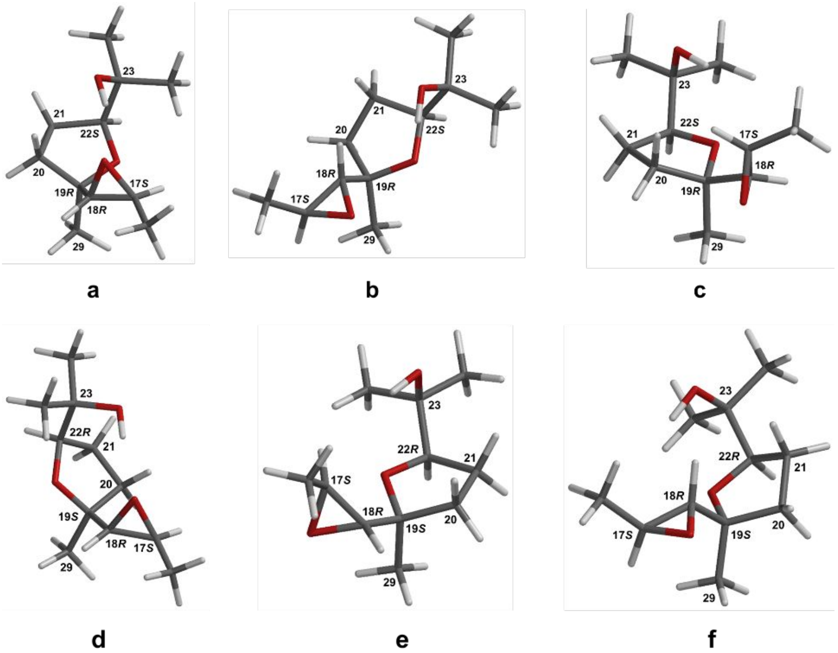

Figure S36. **a**–**c**: Viable conformations of the epoxide with respect to the assumed 19*R*, 22*S* configuration of the tetrahydrofuran ring in peyssobaricanoside D (**18**), **d**–**f**: Viable conformations of the epoxide with respect to the assumed 19*S*, 22*R* configuration of the tetrahydrofuran ring in peyssobaricanoside D (**18**). Considering the computational cost associated with modeling **18** at its entirety, only a portion of **18** as represented by **a**–**f** was subjected to DFT based structural optimization and total nuclear spin-spin coupling *J* (Hz) using B3LYP/6-31G(d,p).

Table S3. Optimized atomic coordinates for conformer **a** (**Figure S36**) using B3LYP/6-31G(d,p)

Atom	x	y	z
O	-0.02269	0.4273	0.94768
C	0.85438	1.17637	0.09031
C	-0.04353	1.72253	-1.04291
H	0.3011	2.69468	-1.40693
H	-0.05873	1.02047	-1.87925
C	-1.43308	1.77771	-0.39572
H	-2.23202	1.69055	-1.13363
H	-1.5764	2.7213	0.14132
C	-1.41427	0.60662	0.60512
C	1.93032	0.23859	-0.44544
C	2.30526	-1.01533	0.22176
C	3.67391	-1.63023	0.09512
H	4.16537	-1.30649	-0.82641
H	4.30099	-1.34364	0.94649
C	1.51623	2.28887	0.91536
H	2.26472	2.83145	0.32753
H	2.0058	1.86555	1.79715
H	0.76475	3.00678	1.25635
H	-1.93969	0.86986	1.53283
C	-2.00288	-0.73271	0.08434
O	-1.40606	-1.0871	-1.16113
H	-0.45123	-1.2206	-1.01241
C	-1.76815	-1.84558	1.12033
H	-2.27142	-1.62734	2.06885
H	-2.15562	-2.79148	0.73139
C	-3.50366	-0.59088	-0.19052
H	-3.69489	0.12316	-0.99549
H	-4.0432	-0.26366	0.70426
H	-3.90877	-1.55714	-0.50336
H	1.73754	-1.27139	1.11474
O	1.4933	-1.02337	-0.98075
H	2.68386	0.73357	-1.06245
H	-0.70129	-1.96311	1.32485
H	3.60558	-2.72284	0.08129

Table S4. Optimized atomic coordinates for conformer **b** (**Figure S36**) using B3LYP/6-31G(d,p)

Atom	x	y	z
O	0.39133	0.56437	-0.79297
C	-0.86044	0.94939	-0.16324
C	-0.44499	1.56047	1.19452
H	-0.31898	2.64333	1.0843
H	-1.1977	1.39307	1.971
C	0.90073	0.88962	1.49592
H	0.75899	-0.09678	1.94797
H	1.53188	1.48349	2.16216
C	1.51136	0.72634	0.09911
C	-1.65152	-0.33727	0.05454
C	-3.11816	-0.4528	0.01073
C	-3.86543	-1.50541	0.79037
H	-3.21963	-2.36127	1.00436
H	-4.23255	-1.09718	1.73851
C	-1.56288	1.93391	-1.09455
H	-2.45132	2.3645	-0.62149
H	-1.86369	1.43399	-2.01779
H	-0.87746	2.7487	-1.34065
H	2.06225	1.63717	-0.18517
C	2.43456	-0.50094	-0.09727
O	1.66527	-1.68801	0.11909
H	0.92361	-1.62514	-0.50285
C	3.00528	-0.50531	-1.52448
H	3.65389	0.36055	-1.69745
H	3.59044	-1.41525	-1.68451
C	3.55972	-0.52013	0.9376
H	3.16015	-0.61079	1.95087
H	4.16358	0.39109	0.87756
H	4.2134	-1.37781	0.7558
H	-3.6928	0.44335	-0.22912
O	-2.30728	-0.91857	-1.08341
H	-1.11527	-1.06801	0.66467
H	2.19676	-0.47329	-2.26029
H	-4.72954	-1.86333	0.22099

Table S5. Optimized atomic coordinates for conformer **c** (**Figure S36**) using B3LYP/6-31G(d,p)

Atom	x	y	z
O	-0.26131	0.20442	0.8338
C	0.86303	0.90815	0.2649
C	0.43506	1.14971	-1.19137
H	0.97447	1.97658	-1.65849
H	0.60566	0.25096	-1.78812
C	-1.08121	1.39398	-1.07228
H	-1.60952	0.99388	-1.93873
H	-1.31076	2.46131	-1.00645
C	-1.49083	0.67093	0.23754
C	2.06514	0.00054	0.48716
C	2.66506	-0.91819	-0.49263
C	3.43844	-2.14033	-0.06554
H	3.82922	-2.01903	0.94837
H	2.79908	-3.0296	-0.09025
C	1.10418	2.22471	1.01979
H	2.01381	2.70805	0.65135
H	1.2214	2.02909	2.08984
H	0.26818	2.91854	0.8909
H	-1.98052	1.37021	0.93051
C	-2.39532	-0.57244	0.06453
O	-1.72797	-1.49651	-0.8023
H	-0.85504	-1.62484	-0.4002

C	-2.65218	-1.23155	1.43053
H	-3.21385	-0.56641	2.09563
H	-3.22917	-2.15005	1.29103
C	-3.72135	-0.21972	-0.60968
H	-3.56726	0.17201	-1.61785
H	-4.27147	0.52439	-0.02487
H	-4.34027	-1.11756	-0.69168
H	2.23186	-0.97143	-1.49142
O	3.27287	0.35712	-0.20061
H	2.20972	-0.24639	1.54249
H	-1.70828	-1.48023	1.92232
H	4.28385	-2.31443	-0.73918

Table S6. Optimized atomic coordinates for conformer **d** (**Figure S36**) using B3LYP/6-31G(d,p)

Atom	x	y	z
C	-0.83103	1.0557	0.25101
O	0.34144	0.76469	1.0438
C	1.55179	0.80748	0.26288
H	2.27632	1.39628	0.84085
C	1.15663	1.52491	-1.04
H	1.74896	1.20182	-1.89768
H	1.29048	2.60572	-0.92449
C	-0.32872	1.17144	-1.20265
H	-0.40948	0.21034	-1.7129
H	-0.88796	1.92133	-1.7692
C	-1.45032	2.35992	0.76888
H	-1.63743	2.29185	1.84472
H	-2.39747	2.57672	0.26268
H	-0.76796	3.19747	0.59859
C	2.1454	-0.61782	0.07564
C	2.199	-1.34879	1.42609
H	2.60663	-2.35334	1.28124
H	1.19804	-1.43075	1.85599
H	2.8307	-0.81712	2.14632
C	3.55021	-0.52296	-0.52959
H	3.96047	-1.52879	-0.65361
H	4.22312	0.04889	0.11718
H	3.52839	-0.05	-1.51527
O	1.3771	-1.37526	-0.86242
H	0.48722	-1.51734	-0.49618
C	-1.79809	-0.09354	0.51087
H	-2.12798	-0.14819	1.5504
C	-2.65733	-0.71883	-0.50627
H	-2.5731	-0.34945	-1.52922
C	-3.96089	-1.39254	-0.16559
H	-3.96505	-1.72754	0.87492
H	-4.12284	-2.26409	-0.8079
H	-4.79839	-0.70256	-0.3159
O	-1.45885	-1.37832	-0.03866

Table S7. Optimized atomic coordinates for conformer **e** (**Figure S36**) using B3LYP/6-31G(d,p)

Atom	x	y	z
C	-0.90757	1.04642	0.07853
O	0.07304	0.11507	0.56633
C	1.38738	0.60213	0.23861
H	1.75713	1.22798	1.06432
C	1.21232	1.4473	-1.04987
H	1.69985	0.9626	-1.89737
H	1.65349	2.44001	-0.92708
C	-0.31485	1.517	-1.26794
H	-0.61576	0.82204	-2.05924
H	-0.66419	2.51383	-1.5508
C	-1.08939	2.19105	1.08581
H	-1.39503	1.77916	2.0502
H	-1.86139	2.88895	0.7451
H	-0.16178	2.7556	1.21995
C	2.30748	-0.63188	0.10528
C	2.32031	-1.4315	1.41869
H	2.92842	-2.33173	1.29304
H	1.30712	-1.72746	1.70198
H	2.7394	-0.84102	2.24075
C	3.72867	-0.22201	-0.28345
H	4.35223	-1.11515	-0.38025
H	4.17171	0.42574	0.47957
H	3.74483	0.30324	-1.24163
O	1.8179	-1.45346	-0.96019
H	0.87692	-1.58281	-0.76792
C	-2.23542	0.33031	-0.11342
H	-2.93562	0.89644	-0.73452
C	-2.41733	-1.12674	-0.03692
H	-1.53974	-1.72028	0.21648
C	-3.50753	-1.84687	-0.78899
H	-4.33727	-1.17159	-1.01574
H	-3.89817	-2.67876	-0.19368
H	-3.12324	-2.25799	-1.72909
O	-2.84135	-0.26662	1.03986

Table S8. Optimized atomic coordinates for conformer **f** (**Figure S36**) using B3LYP/6-31G(d,p)

Atom	x	y	z
C	0.88554	-0.95596	0.29939
O	-0.28163	-0.29079	0.84976
C	-1.49031	-0.69485	0.17228
H	-2.00362	-1.45395	0.78381
C	-1.02021	-1.30764	-1.15073
H	-0.89544	-0.51331	-1.89318
H	-1.72754	-2.03972	-1.54891
C	0.32764	-1.92975	-0.7663
H	1.01513	-2.04417	-1.60884
H	0.17935	-2.9202	-0.32227
C	1.62789	-1.66291	1.42927
H	1.83994	-0.9711	2.24868
H	2.57182	-2.07698	1.0637
H	1.01479	-2.47566	1.82745
C	-2.39371	0.55648	0.06302
C	-2.81579	1.02513	1.46512
H	-3.38652	1.95466	1.38653
H	-1.93648	1.20477	2.09031
H	-3.43829	0.27568	1.96622
C	-3.61868	0.28045	-0.80886
H	-4.25316	1.17045	-0.84576
H	-4.2105	-0.54679	-0.40395

H	-3.32396	0.03449	-1.83206
O	-1.65639	1.59462	-0.59049
H	-0.83474	1.67232	-0.08143
C	1.71601	0.1314	-0.38181
H	1.15891	0.66845	-1.15428
C	2.86166	0.83227	0.21749
H	3.16988	0.54209	1.22222
C	3.25643	2.2263	-0.19927
H	2.89848	2.44695	-1.2086
H	4.34587	2.33594	-0.19165
H	2.8386	2.96762	0.49075
O	3.05786	-0.20846	-0.76256

MicroED Sample Preparation

Sample was prepared according to previously disclosed procedures outlined in Jones *et al*.[4] Data was collected on a Thermo Fisher Talos Arctica F200C transmission electron microscope operating with an accelerating voltage of 200keV, corresponding to an electron wavelength of 0.0251 Å. Electron diffraction data was collected using a Thermo Fisher CetaD camera. Screening the TEM grid for microcrystals was performed at 2600x magnification in imaging mode. Particles were visually selected for data collection and isolated by a selected area aperture. Data was collected by taking images of the diffraction patterns generated by a continuously rotating crystal integrated continuously at a rate of 3 seconds per frame. This rotation was performed at a rate of 0.3° per second with a minimum and maximum tilt range of −70° to +70°. Crystals selected for data collection were isolated by a selected area aperture to reduce the background noise contributions and calibrated to eucentric height to stay in the aperture over the entire tilt range. All diffraction data was processed using the XDS suite of programs as controlled by a custom Python automation script.[5-7] Structures were solved *ab initio* by direct methods in SHELXT or SHELXD and the direct preliminary solution is included for each entry. After this, structures were refined with SHELXL using ShelXle and incorporating electron scattering factors.[8-11] Thermal parameters were refined anisotropically for all non-hydrogen atoms. Hydrogen atoms were assigned using the riding model.

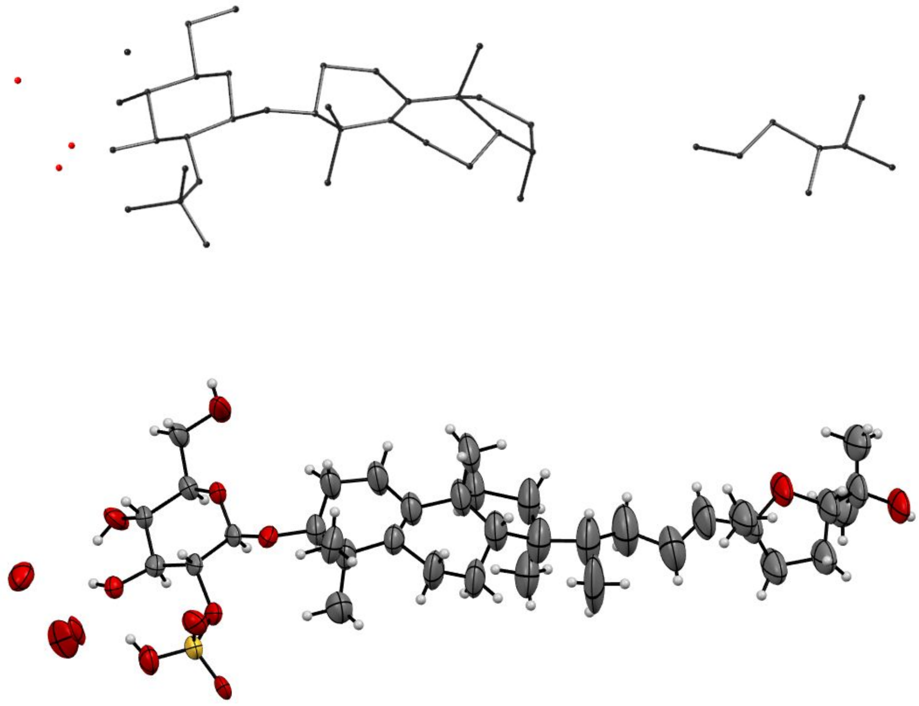

Figure S37. MicroED initial direct methods solution (top) and ORTEP diagram of refined of peyssobaricanoside B (**16**) (bottom). Thermal ellipsoids shown as shaded octants at 30% probability.

Data Collection

Type of instrument	Talos Arctica F200C
Wavelength	0.0215 Å
Data collection temperature	80(4) K
Unit cell dimensions	a = 7.120(10), b = 10.820(2), c = 53.230(4)
Volume	4100.8(10)
Z	4
Crystal system	Orthorhombic
Space group	$P2_12_12_1$
Density (calculated)	1.213 Mg/m^3
F(000)	15
Measured reflections	3383
Reflections with I > 2σ(*I*)	1814
Resolution	1.05 Å
Completeness	89.4%
Index ranges	6 ≤ h ≤ -6, 10 ≤ k ≤ -10, 43 ≤ l ≤ -43

Structure Solution and Refinement

Structure solution program	SHELXD (Uson & Sheldrick, 1999)
Primary solution method	Direct methods
Secondary solution method	Difference Fourier map
Hydrogen placement	Geometric positions
Structure refinement program	SHELXL-2018/3 (Sheldrick, 2018)
Refinement method	Full matrix least-squares on F^2
Data / restraints / parameters	3383 / 466 / 891
Treatment of hydrogen atoms	Riding
Goodness-of-fit on F^2	1.261
Final R indices [I>2s(I)]	R1 = 0.1661, *w*R2 = 0.4087
R indices (all data)	R1 = 0.2351, *w*R2 = 0.4472
Type of weighting scheme used	Sigma
Weighting scheme used	$w=1/s^2(Fo^2)$
Max shift/error	0.000
Average shift/error	0.000
Largest diff. peak and hole	0.19 and -0.14 e.$Å^{-3}$

Special Refinement Details

Refinement of F^2 against ALL reflections. The weighted R-factor (wR) and goodness of fit (S) are based on $F\text{-}^2$, conventional R-factors (R) are based on F, with F set to zero for negative F^2. The threshold expression of $F^2 > 2s(F^2)$ is used only for calculating R-factors(gt) etc. and is not relevant to the choice of reflections for refinement. R-factors based on F^2 are statistically about twice as large as those based on F, and R-factors based on ALL data will be even larger.

All esds (except the esd in the dihedral angle between two l.s. planes) are estimated using the full covariance matrix. The cell esds are taken into account individually in the estimation of esds in distances, angles and torsion angles; correlations between esds in cell parameters are only used when they are defined by crystal symmetry. An approximate (isotropic) treatment of cell esds is used for estimating esds involving l.s. planes.

Given low bond length precision, likely due to poor diffraction data quality, atom identities (C,N,O,S) were assigned during refinement using information gathered from previously performed spectroscopy and mass spectrometry experiments. Twelve C-C single bonds and one C=C double bond distances were forced using DFIX due to distortion of bond lengths, presumably arising from disorder and diffraction quality. This crystal structure is of insufficient quality to unambiguously assign the structure of peyssobaricanoside B (**16**) alone; however, the connectivity of atoms is reliably determined and allows for relative stereochemical assignment in conjunction with other analytical methods.

Microcrystal electron diffraction data of **16** is deposited at CCDC 2251540. These data can be obtained free of charge via http://www.ccdc.cam.ac.uk/conts/retrieving.html, or from the Cambridge Crystallographic Data Centre, 12 Union Road, Cambridge CB2 1EZ, UK; Fax: (+44) 1223-336-033; or Email:deposit@ccdc.cam.ac.uk.

References

1. Norte, M.; Fernández, J.; Souto, M. L. New polyether squalene derivatives from *Laurencia*. *Tetrahedron* **1997**, *53* (13), 4649-4654.
2. Manŕıquez, C. P.; Souto, M. a. L.; Gavı́n, J. A.; Norte, M.; Fernández, J. J. Several new squalene-derived triterpenes from *Laurencia*. *Tetrahedron* **2001**, *57* (15), 3117-3123.
3. Oberg, K. M.; Cochran, B. M.; Cook, M. J.; Rovis, T. The Catalytic Alkylative Desymmetrization of Anhydrides in a Formal Synthesis of Ionomycin. *Synthesis.* **2018**, *50* (22), 4343-4350.
4. Jones, C. G.; Martynowycz, M. W.; Hattne, J.; Fulton, T. J.; Stoltz, B. M.; Rodriguez, J. A.; Nelson, H. M.; Gonen, T. The CryoEM method MicroED as a powerful tool for small molecule structure determination. *ACS Central Science* **2018**, *4* (11), 1587-1592.
5. Kabsch, W. XDS. *Acta Cryst.* **2010**, *D66*, 125-132.
6. Kabsch, W. Integration, scaling, space-group assignment and post-refinement. *Acta Cryst.* **2010**, *D66*, 133-144.
7. Hattne, J.; Reyes, F. E.; Nannenga, B. L.; Shi, D.; de la Cruz, M. J.; Leslie, A. G.; Gonen, T. MicroED data collection and processing. *Acta Cryst.* **2015**, *A71*, 353-360.
8. Sheldrick, G. M. A short history of SHELX. *Acta Crystallogr A* **2008**, *64* (1), 112-122.
9. Sheldrick, G. M. SHELXT - integrated space-group and crystal-structure determination. *Acta Cryst.* **2015**, *A71*, 3-8.
10. Sheldrick, G. M. SHELXT - integrated space-group and crystal-structure determination. *Acta Cryst.* **2015**, *C71*, 3-8.
11. Hübschle, C. B.; Sheldrick, G. M.; Dittrich, B. ShelXle: a Qt graphical user interface for SHELXL. *J. Appl. Crystallogr.* **2011**, *44* (6), 1281-1284.
